# Comparative Thermal Performance of Ultra-High-Performance Concrete and Geopolymer Concrete: Influence of Steel Fibre Geometry on Residual Mechanical and Chemical Properties

**DOI:** 10.3390/ma19163562

**Published:** 2026-08-21

**Authors:** Yusra Muhammed, Jawdat Tashan, Nadia Saiyouri, Youssef Sleiman, Bland Lateef

**Affiliations:** 1 Department of Architecture with Construction, School of Engineering and Technology, British International University, Erbil 44001, Iraq; yusra.muhammed@britishuniversity.krd; 2 Department of Civil Engineering, School of Science and Engineering, University of Kurdistan-Hewler, Erbil 44001, Iraq; jawdat.tashan@ukh.edu.krd (J.T.); b.lateef3@ukh.edu.krd (B.L.); 3 Department of Civil and Environmental Engineering, University of Bordeaux, 33405 Talence Cedex, France; nadia.saiyouri@u-bordeaux.fr

**Keywords:** Ultra-High-Performance Geopolymer Concrete (UHPGC), Ultra-High-Performance Concrete (UHPC), fibre-reinforced cementitious composites, elevated temperature exposure, steel fibre, materials design

## Abstract

To investigate the elevated-temperature performance of Ultra-High-Performance Concrete (UHPC) and Ultra-High-Performance Geopolymer Concrete (UHPGC), a systematic comparative study was conducted at 800 °C. This study examined the effects of the steel fibre geometry (micro and hooked-end) and dosage (1.5% and 2.0%) on mass loss, crack propagation, residual compressive, flexural, and tensile strengths, and chemical evolution following a 24 h pre-drying protocol to mitigate explosive spalling. The results demonstrate that UHPGC exhibits superior thermal stability and residual mechanical performance compared with UHPC after high-temperature exposure. Among all mixtures, the UHPGC mixture reinforced with 2% micro steel fibres (UHPGC-M2) achieved the highest residual compressive strength (30 ± 0.4 MPa, corresponding to 25% strength retention compared with 21% for the equivalent UHPC mixture), the lowest post-exposure crack width (0.08 mm), and the highest tensile strength retention (17.9%). Micro steel fibres were more effective in controlling crack propagation and preserving peak load capacity, whereas hooked-end fibres contributed more significantly to post-peak ductility. Chemical analysis revealed substantial chemical changes in both systems after exposure to 800 °C. However, UHPGC exhibited lower mass loss (4.8%) and greater residual performance. These findings establish micro steel fibre-reinforced UHPGC as a sustainable and high-performance material for fire-resistant structural applications.

## 1. Introduction

Ultra-High-Performance Concrete (UHPC) is a cementitious composite widely recognised for its exceptional mechanical strength, durability, and superior performance, making it increasingly suitable for advanced structural applications [1,2]. Continuous research efforts have been devoted to optimising the mix design of UHPC to achieve enhanced performance while reducing material consumption. Statistical mixture design approaches have been successfully employed to predict compressive and flexural strength while minimising the binder content [1]. In addition, well-established design principles, such as eliminating coarse aggregates, optimising the particle packing density, and incorporating a high silica fume content, contribute to the development of dense, high-strength matrices [3]. Standardised guidelines further provide recommendations on curing conditions, fibre incorporation, and testing procedures to ensure consistent UHPC performance [4].

Despite these advantages, UHPC is highly vulnerable when exposed to elevated temperatures or fire conditions. The dense microstructure and low permeability, which are beneficial for durability, hinder moisture migration and lead to the buildup of internal vapour pressure [5,6]. This phenomenon significantly increases the risk of explosive spalling, primarily driven by rapid pore-pressure development and thermal stresses within the matrix [6,7]. Furthermore, exposure to high temperatures accelerates shrinkage and induces microcracking, altering the pore structure and degrading residual mechanical properties [7]. When temperatures exceed 200 °C, substantial microstructural changes occur. These changes, including the decomposition of the calcium silicate hydrate (C–S–H) gel and increased porosity, ultimately compromise the structural integrity of UHPC [8,9,10].

Several studies have investigated the behaviour of UHPC under elevated-temperature conditions. Phan et al. [11] reported that specimens with a water-to-cement ratio of 0.33 remained stable at 450 °C. In contrast, those with a ratio of 0.22 exhibited severe spalling at the same temperature, highlighting the importance of mixture design for thermal performance. Chan et al. [12] observed that high-performance concrete specimens subjected to 800 °C exhibited a higher reduction in compressive strength, approximately 34% higher than that of normal-strength concrete. Due to its dense microstructure, UHPC typically exhibits greater strength degradation under similar conditions [5,7]. Moreover, the residual mechanical properties of UHPC are influenced by factors such as the mixture composition, fibre content, and specimen characteristics [10,13]. Previous studies have reported substantial reductions in mechanical performance within the temperature range of 200 °C to 800 °C [13]. Interestingly, a temporary increase in compressive strength of up to 30% has been observed at temperatures between 300 °C and 400 °C due to continued hydration and densification [14,15,16]. However, this trend reverses beyond 500 °C, where residual strength typically decreases by 15–25% compared to ambient conditions [17]. While compressive strength behaviour has been extensively studied, limited research has focused on the post-fire flexural and tensile performance of UHPC, particularly in steel fibre-reinforced systems [10].

To address these limitations, fibre reinforcement has been widely adopted to enhance UHPC’s fire resistance. Liang et al. [14] demonstrated that UHPC incorporating steel-slag powder and hybrid steel–polypropylene fibres retained approximately 69% of its original compressive strength after exposure to 800 °C. Similarly, Xiong and Liew [9] reported that steel fibre reinforcement effectively reduced spalling and enabled UHPC to retain nearly 70% of its compressive strength after high-temperature exposure. Other studies have also confirmed that hybrid fibre systems can improve crack resistance and enhance thermal stability, thereby mitigating the adverse effects of elevated temperatures [10,17].

However, the extensive use of Ordinary Portland Cement (OPC) in UHPC raises significant environmental concerns. Cement production contributes approximately 7% of global carbon dioxide (CO_2_) emissions, necessitating the development of sustainable alternative materials [18,19]. In this context, Ultra-High-Performance Geopolymer Concrete (UHPGC) has emerged as a promising substitute. Geopolymer binders, derived from industrial by-products such as fly ash, ground granulated blast-furnace slag, and metakaolin, offer a reduced carbon footprint while maintaining comparable mechanical performance [20]. Several studies have demonstrated that geopolymer concrete exhibits superior thermal resistance compared to conventional cement-based systems. For instance, fly ash- and metakaolin-based geopolymer concretes have shown stable mechanical performance even after exposure to temperatures up to 1000 °C [21]. Zhao et al. [22] observed that fly ash-based geopolymer concrete retained its structural integrity up to 300 °C, exhibiting minimal microstructural deterioration and enhanced residual compressive strength. Haider et al. [23] reported that the combined use of metakaolin and silica fume enhanced fire resistance and improved residual strength retention after exposure to elevated temperatures.

Furthermore, the incorporation of fibres into geopolymer matrices has been shown to remarkably enhance post-fire performance. Studies indicate that steel fibre reinforcement reduces thermal cracking and improves residual strength compared to non-reinforced geopolymer concrete [24]. The inclusion of steel fibres has also been found to prevent explosive spalling and improve mechanical retention after exposure to 800 °C [25]. For example, the use of 1% hooked steel fibres enabled fly-ash-based geopolymer concrete to retain approximately 30% of its compressive strength while minimising spalling effects [25]. Nevertheless, the optimal type, geometry, and dosage of steel fibres in geopolymer systems remain insufficiently explored.

In recent years, increasing attention has been directed toward understanding the effects of elevated temperatures on both UHPC and UHPGC, particularly in fire-prone structural applications. Although previous studies have highlighted the importance of fibre reinforcement in enhancing thermal resistance and residual strength, a comprehensive comparison between UHPC and UHPGC at ultra-high strength levels remains lacking. Specifically, limited research has addressed the influence of the fibre geometry and optimised fibre dosage on post-fire mechanical performance [5,8,11,19]. Existing studies report a broad range of material configurations and thermal performances for UHPC and UHPGC, as shown in Table A1. This leads to inconsistent trends in strength retention and damage mechanisms. While Table A1 is intended to contextualise the current state of knowledge, it also provides a focused comparison with the results obtained in this study. The analysis is deferred to the concluding Discussion section, where similarities, discrepancies, and contributions are critically evaluated [26,27,28,29,30,31,32,33,34,35,36,37,38,39,40,41,42,43,44,45,46].

Therefore, this study aims to compare the mechanical performance of UHPC and UHPGC reinforced with hooked-end and micro steel fibres under elevated temperatures up to 800 °C by evaluating residual compressive, flexural, and splitting tensile strengths. Emphasis is placed on thermal degradation behaviour, chemical changes (XRF), and the influence of the fibre geometry and dosage on post-fire mechanical performance and stability. Despite growing interest in UHPC and UHPGC, direct comparative studies at the ultra-high-performance level under identical experimental conditions remain scarce. This study provides new insights into the influence of the fibre geometry and dosage on the post-fire mechanical performance and thermal stability of UHPC and UHPGC.

### 1.1. Research Objectives

The primary objective of this study is to evaluate and compare the post-fire mechanical performance and chemical evolution of UHPC and UHPGC reinforced with different steel fibre geometries under controlled high-temperature exposure conditions. The study specifically aims to

(a) To evaluate the residual compressive, flexural, and splitting tensile strengths of UHPC and UHPGC reinforced with hooked-end and micro steel fibres before and after exposure to temperatures up to 800 °C;
(b) To evaluate thermal deterioration, including mass loss, surface damage, and spalling resistance, through physical measurements and visual inspection following high-temperature exposure;
(c) To investigate chemical changes in UHPC and UHPGC matrices using X-ray fluorescence (XRF) to identify temperature-induced variations and degradation mechanisms;
(d) To assess the influence of fibre geometry and dosage on post-fire mechanical performance, indirectly inferred microstructural integrity, and the chemical stability of ultra-high-performance cementitious composites.

### 1.2. Research Limitations

The present study was limited by the absence of advanced microstructural characterisation techniques, such as SEM, the use of a single maximum exposure temperature (800 °C), and the exclusion of hybrid fibre systems and nano-additives. These limitations provide directions for future research aimed at improving fire resistance and residual mechanical performance.

## 2. Experimental Program

### 2.1. Materials

#### 2.1.1. Powder Materials

Ordinary Portland Cement (CEM I 42.5 R SR5) was used in the manufacture of Ultra-High-Performance Concrete (UHPC). Ultra-High-Performance Geopolymer Concrete (UHPGC) was made from Natural Pozzolan Fly Ash (FA, Class N) and Ground Granulated Blast Furnace Slag (GGBS). The selection of these SCMs was based on their very high pozzolanic reactivity, enhanced fire resistance, and reduced environmental impact compared to OPC [1,5].

Both GGBS and FA are produced through very high-temperature manufacturing processes. GGBS is a by-product generated from iron ore in a blast furnace (operating at about 1500 °C with coke, limestone, and iron ore) to create iron, while FA is produced by combusting coal (at about 1400 °C). They can both react with lime and water, thereby developing cementitious properties, making them ideally suited as binders in geopolymer concrete.

Table 1 summarises the chemical and physical properties of OPC, GGBS, and FA, and the physical appearance of GGBS and FA used in the production of UHPGC. Local silicate with particle sizes ranging from 0.1 to 2 mm was used as the aggregate in all concrete mixtures.

#### 2.1.2. Steel Fibres

The UHPC and UHPGC mixtures were reinforced with different fibre types and dosages. Two types of steel fibres were incorporated into the concrete mixtures: micro steel fibres and hooked-end steel fibres. These fibres were added to enhance the mechanical performance and structural integrity of the concrete, particularly under elevated-temperature conditions. Steel fibres improve the tensile capacity, crack-bridging ability, and post-cracking behaviour of UHPC, while maintaining performance under elevated temperatures due to their superior thermal stability [47]. Steel fibres have a high melting point, making them suitable for fire-exposed applications [5]. The physical and mechanical properties of the steel fibres, as provided by the manufacturer, are presented in Table 2. At the fibre dosages used in this study, the addition of steel fibres increases the material cost by approximately €22.3/m^3^ at a 1.5% volume fraction and €29.7/m^3^ at a 2.0% volume fraction, based on a fibre cost of €1500/ton. While this represents a modest absolute increase relative to typical UHPC material costs, steel fibres remain a significant cost driver in UHPC formulations more broadly. This added cost should be weighed against the improved thermal resistance and residual mechanical performance demonstrated in Section 3.

#### 2.1.3. Chemical Admixtures and Alkaline Activator

To ensure uniformity, reliability, and international comparability, all specimen preparation and testing procedures adhered to relevant ASTM standards. A polycarboxylate-based superplasticiser was employed to improve the workability and flowability of the fresh concrete mixtures. Sika ViscoCrete-TEMPO-10 (Ogun State, Nigeria) was used as the chemical admixture and is classified as a Type F high-range water-reducing admixture according to ASTM C494 [48]. For UHPGC production, an alkaline activator solution was prepared using sodium silicate (Na_2_SiO_3_) and sodium hydroxide (NaOH) [49]. Sodium silicate was supplied in liquid form, while sodium hydroxide was provided as pellets.

### 2.2. Mix Design and Casting Procedures

**Figure 1:**
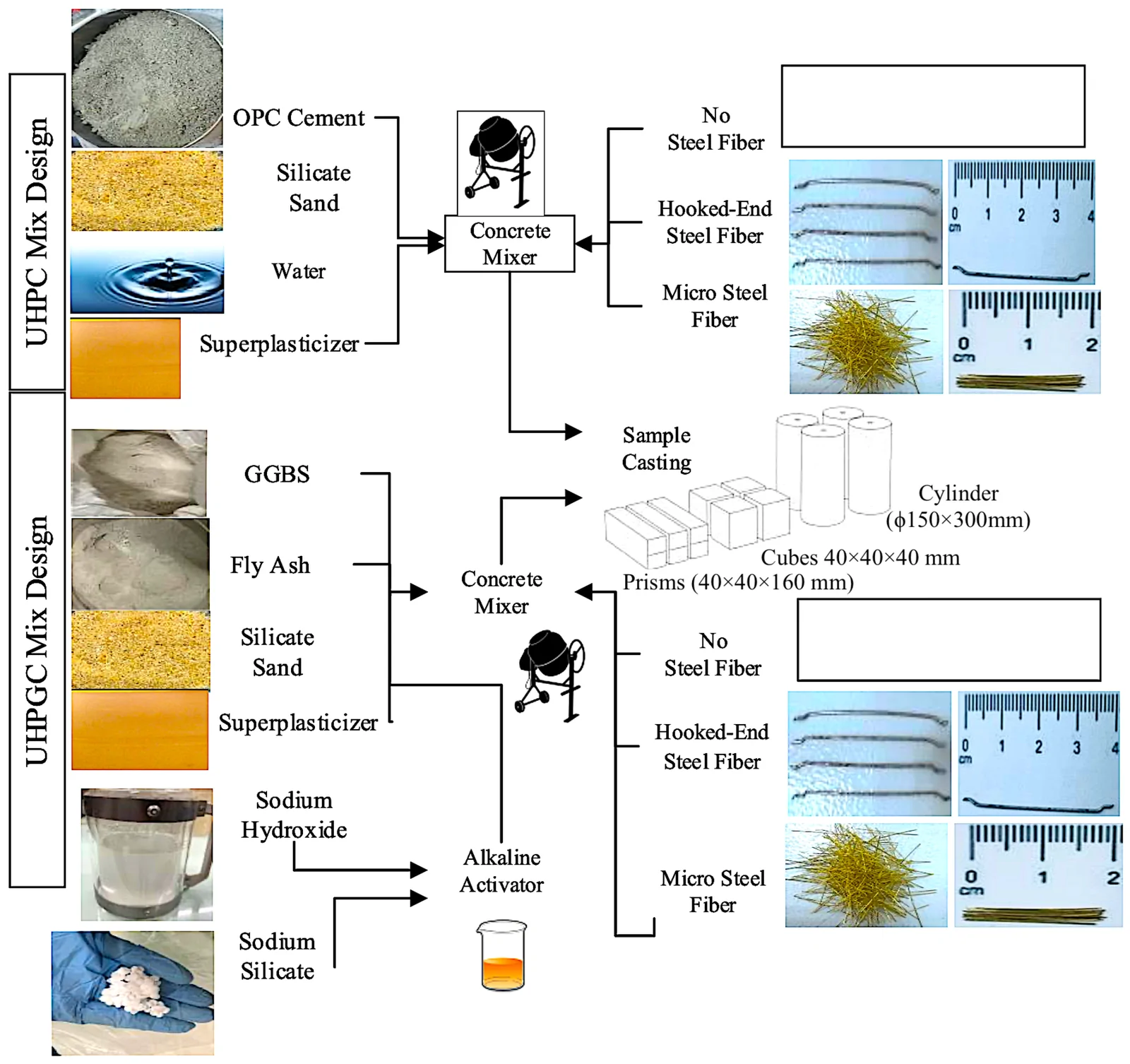
Materials and components used in the preparation of the concrete mixtures.

UHPC and UHPGC mix designs were optimised in accordance with ASTM C1856 [50] through a series of preliminary trials. Based on these trials, steel fibre contents of 1.5% and 2% were selected for the main experimental program due to their superior post-fire mechanical performance. The detailed mix proportions and fibre dosages are presented in Table 3. Also, the summary of the materials is shown in Figure 1.

For UHPGC, GGBS, and FA, the incorporation rates were 60% and 40% of the total binder mass, respectively. This combination was selected to balance the high calcium content of GGBS, which promotes early strength development, with the aluminosilicate-rich and pozzolanic nature of FA, which improves workability, long-term strength, and durability. Previous studies have demonstrated that blended GGBS–FA geopolymer systems exhibit enhanced mechanical and thermal properties [21,25].

To obtain a 10 M NaOH solution, 400 g of NaOH pellets (100% purity) were dissolved in 1 L of distilled water. The solution was prepared 24 h before mixing to allow complete dissolution, stabilise the temperature, and minimise exothermic effects. Sodium hydroxide and sodium silicate solutions were prepared separately and combined during casting. The final alkaline activator solution was thoroughly mixed and allowed to rest for 3 h before use.

For UHPGC mixtures, dry mixing was performed for 1 min, followed by the addition of liquids (water, alkaline activator solution, and superplasticiser) and 5 min of mixing at 60 rpm. Steel fibres were subsequently added at the specified dosage, and mixing continued for an additional 5 min at 120 rpm. The workability of all mixtures was deemed satisfactory based on flow table tests.

**Figure 2:**
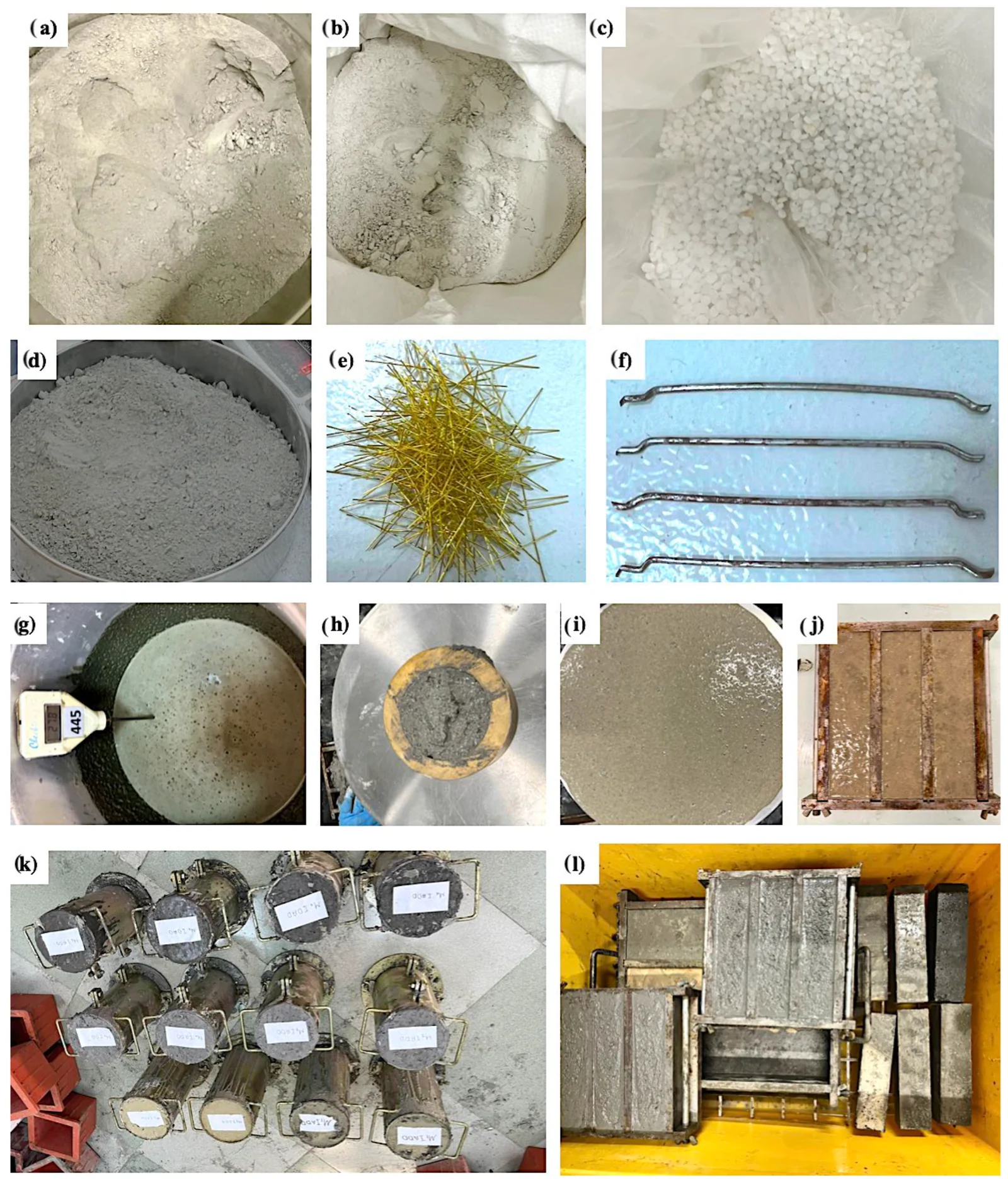
Sample casting procedures: (**a**) fly ash, (**b**) GGBS, (**c**) NaOH pellets, (**d**) cement, (**e**) micro steel fibre, (**f**) hooked-end fibre, (**g**) evaluation of mixture temperature, (**h**) setup of flow test, (**i**) flow table test, (**j**) prism samples, (**k**) cylindrical samples, and (**l**) designed curing chamber.

After casting and surface finishing, the specimens were cured in a controlled-environment chamber at 15 °C and 90% relative humidity. This curing regime was adopted to accelerate hydration and geopolymerisation processes and to promote the development of a dense microstructure. After 28 days of curing, specimens were subjected to mechanical, physical, and thermal testing. Figure 2 illustrates the mixing, casting, and curing processes.

### 2.3. Heating Samples

To minimise uncontrolled explosive spalling caused by free water, specimens were oven-dried at 105 °C for 24 h before the controlled furnace program. This pre-drying step is commonly used in laboratory thermal stability studies [51], focusing on changes in the binder/fibre matrix rather than on evaluating spalling under realistic fire loads. After drying, the specimens were subjected to controlled thermal degradation in a furnace. A heating rate of 5 °C/min was used during the procedure.

**Figure 3:**
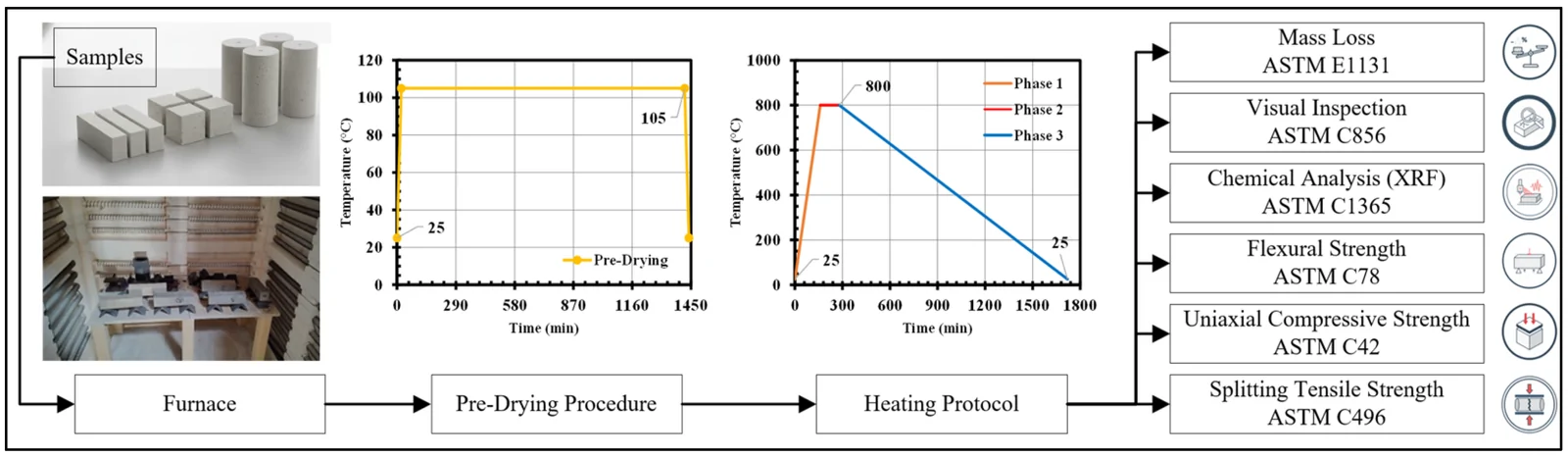
Heating procedures and subsequent testing methods.

The thermal treatment protocol consisted of three phases, as shown in Figure 3. Phase 1, lasting 160 min, involved heating the specimens to 800 °C. Phase two maintained a constant temperature of 800 °C for 120 min to simulate prolonged exposure. Phase three consisted of a controlled cooling process, during which the specimens were gradually cooled to ambient room temperature over 24 h. All mechanical and physical tests were conducted only after the specimens had thoroughly cooled to room temperature. The sample setup arrangement within the furnace is shown in Figure 3.

### 2.4. Testing at Post-Fire Condition

#### 2.4.1. Mass Loss Measurements

To test the effects of high-temperature exposure, the mass loss of each specimen was measured before and after exposure using a laboratory electronic balance, in accordance with ASTM E1131-20 [52]. Mass loss is an essential measure of thermal tolerance that determines the structural integrity of the concrete matrix. The mass loss was calculated by subtracting the post-exposure dry weight from the initial oven-dried weight of each prism-sized specimen, ensuring that the moisture content did not influence the results. A total of 100 prism-sized specimens were utilised to determine the mass loss of the developed mixtures under thermal exposure. However, no thermocouples or sensors were attached to measure real-time mass loss during heating.

(1) $$\%ML=\frac{{W_{i}-W}_{f}}{W_{i}}\times 100$$

where %ML is the percentage mass loss, $W_{i}$ is the initial oven-dried weight of the specimen before exposure to high temperatures, and, $W_{f}$ is the post-exposure dry weight of the specimen.

During measurement, the specimens were visually examined for any changes resulting from exposure to temperatures up to 800 °C, in accordance with ASTM C856-23 [53]. Using magnifying tools and precise lighting, the cracks were examined. This inspection aims to evaluate the effects of high-temperature exposure on concrete and the resulting modifications. All formulated specimens were visually inspected before and after exposure to high temperatures.

#### 2.4.2. Oxide Composition and Chemical Characterisation

The chemical composition of the specimens was determined before and after exposure to 800 °C in accordance with ASTM C1365-18 [54]. The analysis was conducted to evaluate temperature-induced changes in the chemical composition of the developed fibre-reinforced cementitious composites and to support the interpretation of their thermal degradation behaviour. Chemical characterisation was performed using X-ray fluorescence (XRF) (Akishima, Tokyo, Japan), which determines the elemental oxide composition rather than the presence of crystalline or amorphous phases. Therefore, changes in measured oxide concentrations after thermal exposure are interpreted as evidence of chemical redistribution and phase transformation within the matrix rather than direct elemental loss.

#### 2.4.3. Mechanical Testing

A total of 100 specimens, each measuring 40 mm × 40 mm × 160 mm, were tested using the three-point load test method as specified in ASTM C78/C78M-23 [55]. This method involved loading the specimens at 0.5 mm/min and measuring deflection at the centre of each specimen.

The uniaxial compression test was conducted in accordance with ASTM C42/C42M-21 [56]. After flexural strength testing, the identical prism-shaped specimens were cut into cube-shaped samples and subsequently used for uniaxial compression testing. The cubes measured 40 mm× 40 mm × 40 mm. The axial compressive load was applied to these 200 developed specimens at high temperature, both before and after exposure, and the specimens were tested until failure.

The splitting tensile strength of cylindrical specimens was measured following EN 12390-6:2023 [57]. Tensile stress was uniformly distributed along the vertical axis of 40 cylindrical specimens (ϕ150 × 300 mm). For each formulated mixture, four specimens were examined, comprising two tested before and two after exposure to elevated temperatures. It was subjected to a load along its entire length during the test and eventually failed. The specimens were evaluated before and after high-temperature exposure to determine the concrete’s tensile behaviour.

## 3. Results and Discussion

### 3.1. Experimental Results

#### 3.1.1. Mass Loss

**Figure 4:**
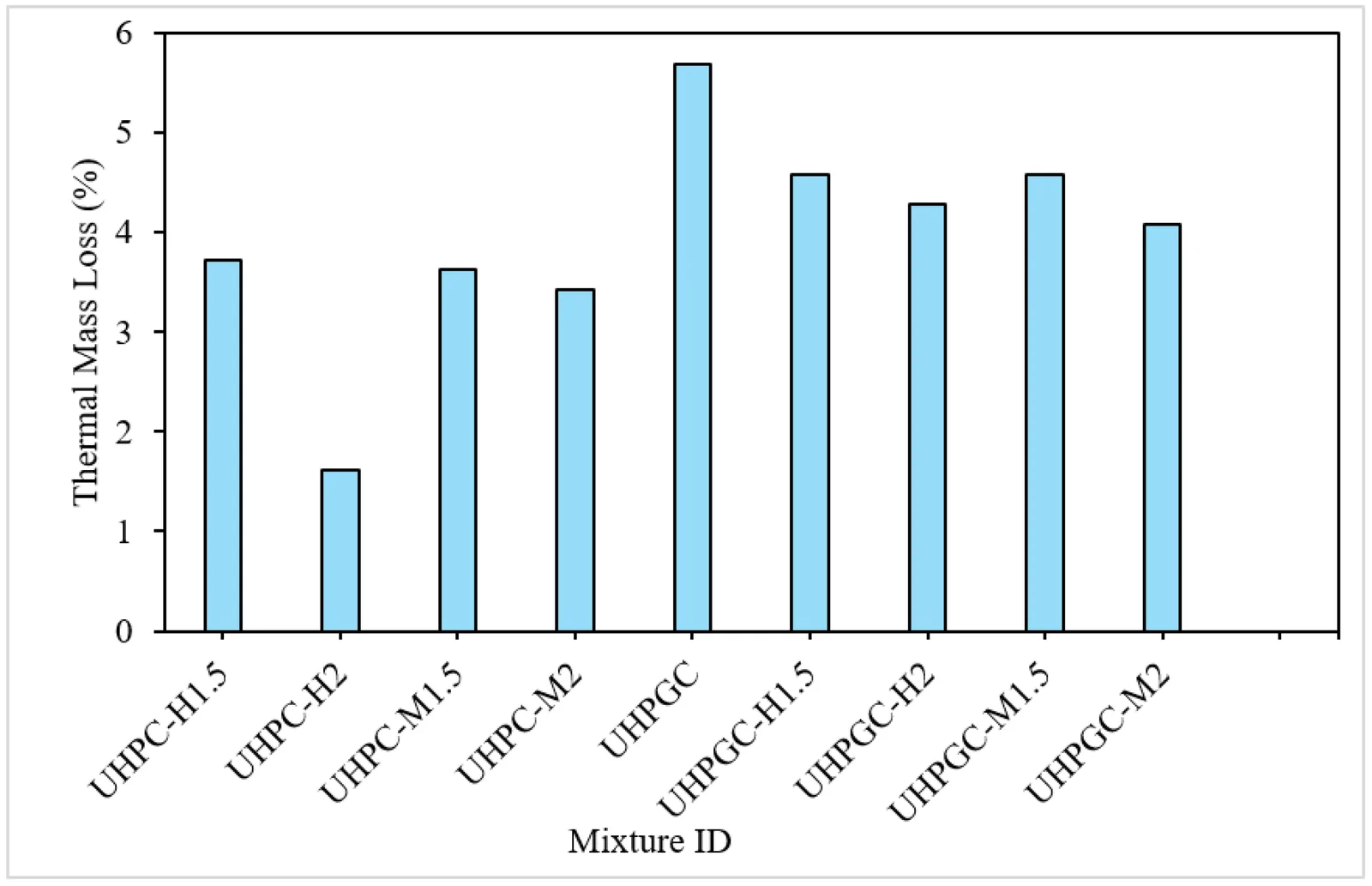
Corrected thermal mass loss (%) of the formulated mix designs after exposure to 800 °C.

The mass-loss test was conducted to evaluate the thermal resistance of UHPC and UHPGC mixtures after exposure to 800 °C, and the corrected thermal mass-loss results are presented in Table 4 and Figure 4. The specimens were exposed to fire conditions and allowed to cool before post-heating mass loss was measured. Apparent differences were observed among the concrete designs, underscoring the critical role of fibre inclusion and dosage. The plain UHPC exhibited the highest mass loss of 6.2%, indicating substantial thermal degradation and matrix deterioration in the absence of fibre reinforcement. In contrast, fibre-reinforced UHPC mixes with increasing fibre contents demonstrated progressively lower mass loss. UHPC-H2 exhibited the lowest loss at 3.0%, followed by UHPC-M2 at 5.0% and UHPC-H1.5 at 5.1%, respectively. A similar trend was observed in UHPGC mixtures. The unreinforced UHPGC mix exhibited a slightly higher mass loss of 6.4% than UHPC, likely due to differences in binder chemistry and decomposition mechanisms. Fibre-reinforced UHPGC mixes exhibited enhanced thermal stability, with UHPGC-M2 achieving the lowest mass loss at 4.8%, followed by UHPGC-H2 and UHPGC-M1.5 at 5.0% and 5.3%, respectively.

The mass-loss results were adjusted to account for binder properties and fineness. Table 4 demonstrates the thermal mass-loss percentages after correcting for these factors, providing a more precise estimate of the actual thermal degradation of the concrete matrix. The UHPC thermal decomposition loss reduced to 4.82% after accounting for the LOI of OPC and the fineness of the cement powder. UHPGC plain showed an adjusted thermal mass loss of 5.68% due to its lower LOI and slightly finer binder combination.

Similar adjustments were applied to all fibre-reinforced mixtures, showing that mass loss depends on the fibre type, dosage, and binder properties. UHPC-H1.5 and UHPC-H2 decreased from 5.1% and 3% to 3.72% and 1.62%, respectively, while UHPC-M1.5 and UHPC-M2 dropped from 5% and 4.8% to 3.62% and 3.42%, respectively. In UHPGC, H1.5, H2, M1.5, and M2 adjusted from 5.3%, 5%, 5.3%, and 4.8% to 4.58%, 4.28%, 4.58%, and 4.08%, respectively. These corrections highlight the combined influence of fibre reinforcement, binder composition, LOI, and fineness on thermal stability.

These findings indicate that 2% of micro steel fibres provide superior crack bridging at the microstructural level, thereby limiting mass loss under extreme thermal conditions. UHPGC mixtures generally exhibit higher residual mass retention under the same thermal exposure. This is due to the inherent thermal stability of the geopolymer matrix, which forms a dense microstructure. In contrast, UHPC relies on C-S-H gels that decompose at high temperatures. The UHPGC specimens weighed less than the UHPC specimens before exposure to high temperatures. This shows differences in the binder density and composition. However, after LOI and fineness adjustments, UHPGC retained more thermal mass (5.68%) than UHPC (4.82%), indicating better thermal stability despite its lower initial mass.

The reduction in mass loss is attributed to fibre reinforcement, which restrains thermally induced microcracks and improves its resistance [58]. Nonetheless, fibres cannot entirely prevent mass loss at temperatures above 600 °C, where dehydration of the C-S-H gel and matrix decomposition dominate [11,58]. The pre-treatment of specimens in a dry oven at 105 °C for 24 h before furnace exposure effectively prevented explosive spalling by gradually removing internal moisture. While this method ensures reproducible laboratory results, it limits real-world applicability, highlighting the need for further studies under uncontrolled fire conditions.

Compared with prior studies, the present mixtures exhibited lower mass loss, due to a combination of the optimised fibre dosage and type, enhanced curing, and the inherent thermal stability of the geopolymer matrix. Similar improvements in post-fire residual properties and reduced cracking with the incorporation of polypropylene fibre were reported by Eidan et al. [59]. The exceptional performance of UHPGC-M2 can be ascribed to its aluminosilicate-based matrix, which is inherently more thermally stable than the calcium-rich OPC matrix of UHPC. Additionally, the higher surface area and random dispersion of 2% micro steel fibres improved crack control compared to hooked-end fibres, thereby minimising mass loss. At temperatures below 800 °C, the developed mixtures exhibited lower mass loss than previously reported values, including those of Liang et al. [14]. A small trial assessed spalling in specimens that were not pre-dried. These specimens spalled at 800 °C, highlighting that pre-treatment mitigates explosive failure. This limitation should be considered when applying these laboratory results to real-world fire conditions. These results show that the binder chemistry, fibre geometry, and pre-treatment protocols interactively control mass loss at high temperatures.

UHPC has 66.5% CaO in its composition, compared to 27.1% in UHPGC, which explains UHPC’s higher susceptibility to thermal decomposition, as Ca-based hydrates decompose at elevated temperatures [11]. Conversely, UHPGC relies more on aluminosilicate gel formation from GGBS and fly ash, which retains the structure better at high temperatures, providing enhanced thermal stability. After accounting for the LOI and fineness effect, the adjusted mass loss indicates that thermal degradation is lower than the experimental values suggest. The finer OPC in UHPC accelerates heat transfer and contributes slightly to mass loss, while the lower LOI of UHPGC binders reduces the mass lost from pre-existing volatiles. Therefore, the combination of lower LOI, fineness, and optimised fibre reinforcement explains the superior thermal stability of UHPGC mixtures.

#### 3.1.2. Visual Appearance and Crack Pattern

**Figure 5:**
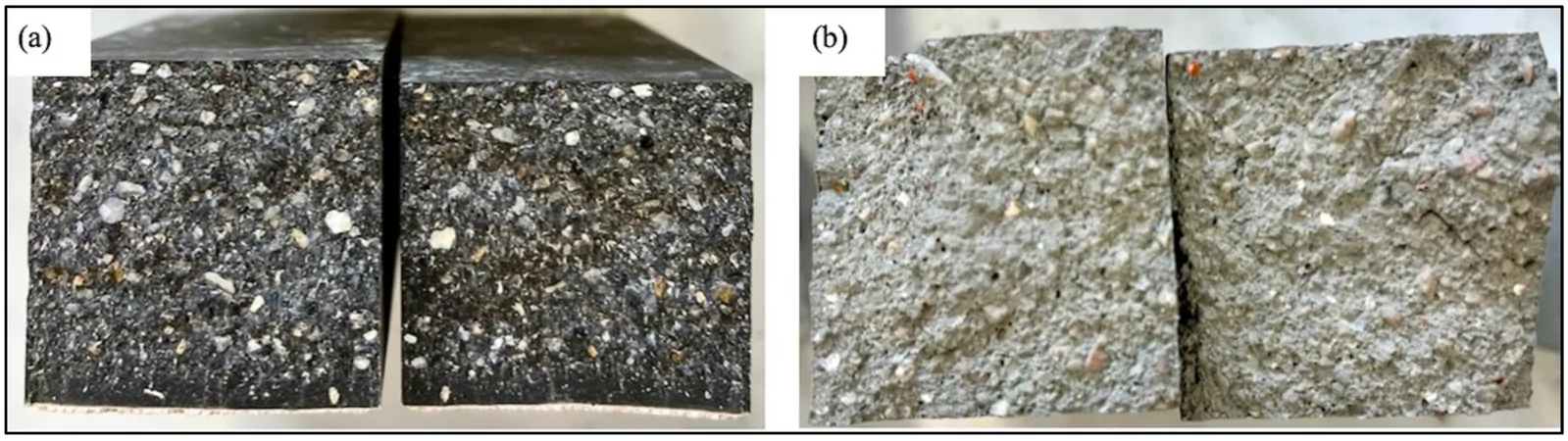
Cross-section of UHPC specimens after flexural failure to detect internal colour changes. (**a**) Before exposure to 800 °C, and (**b**) after exposure to 800 °C.

Exposure to high temperatures resulted in noticeable changes in the colour of the concrete specimens. Initially, the UHPC specimen appeared dark grey at room temperature. However, after exposure at 800 °C, the UHPC specimen’s colour shifted to a lighter grey. This colour alteration indicates substantial changes in both the surface and internal composition. This results from heat-induced chemical processes, such as the dehydration of the cement paste. This is illustrated in Figure 5a,b. Aggregate particles undergo mineral transformations when exposed to high temperatures. As shown in Figure 5b, the appearance of an orange hue indicates the oxidation of specific minerals due to prolonged thermal exposure.

**Figure 6:**
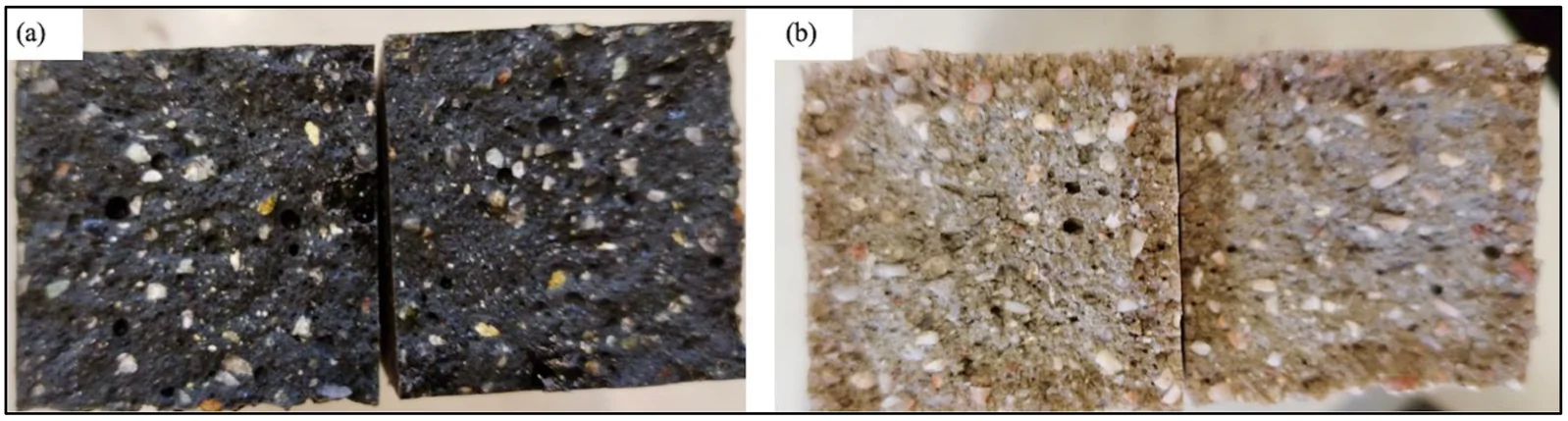
Cross-section of UHPGC specimens after flexural failure to detect internal colour changes. (**a**) Before exposure to 800 °C, and (**b**) after exposure to 800 °C.

Similarly, the UHPGC mixtures also exhibited colour alterations, transitioning from dark grey to lighter grey, as shown in Figure 6a,b. As shown in Figure 6b, the colour change occurs gradually, starting from the core of the concrete and spreading outward toward the edges. Thus, the internal edges are light brown, while the core remains light grey. The orange colour development at the edges of UHPGC specimens, as shown in Figure 6b, is due to the development of iron oxide in the concrete composition. The discolouration and development of surface cracks are key indicators of changes in the structural integrity, primarily due to chemical reactions in aggregates, fibre reinforcements, and cementitious materials at high temperatures.

**Figure 7:**
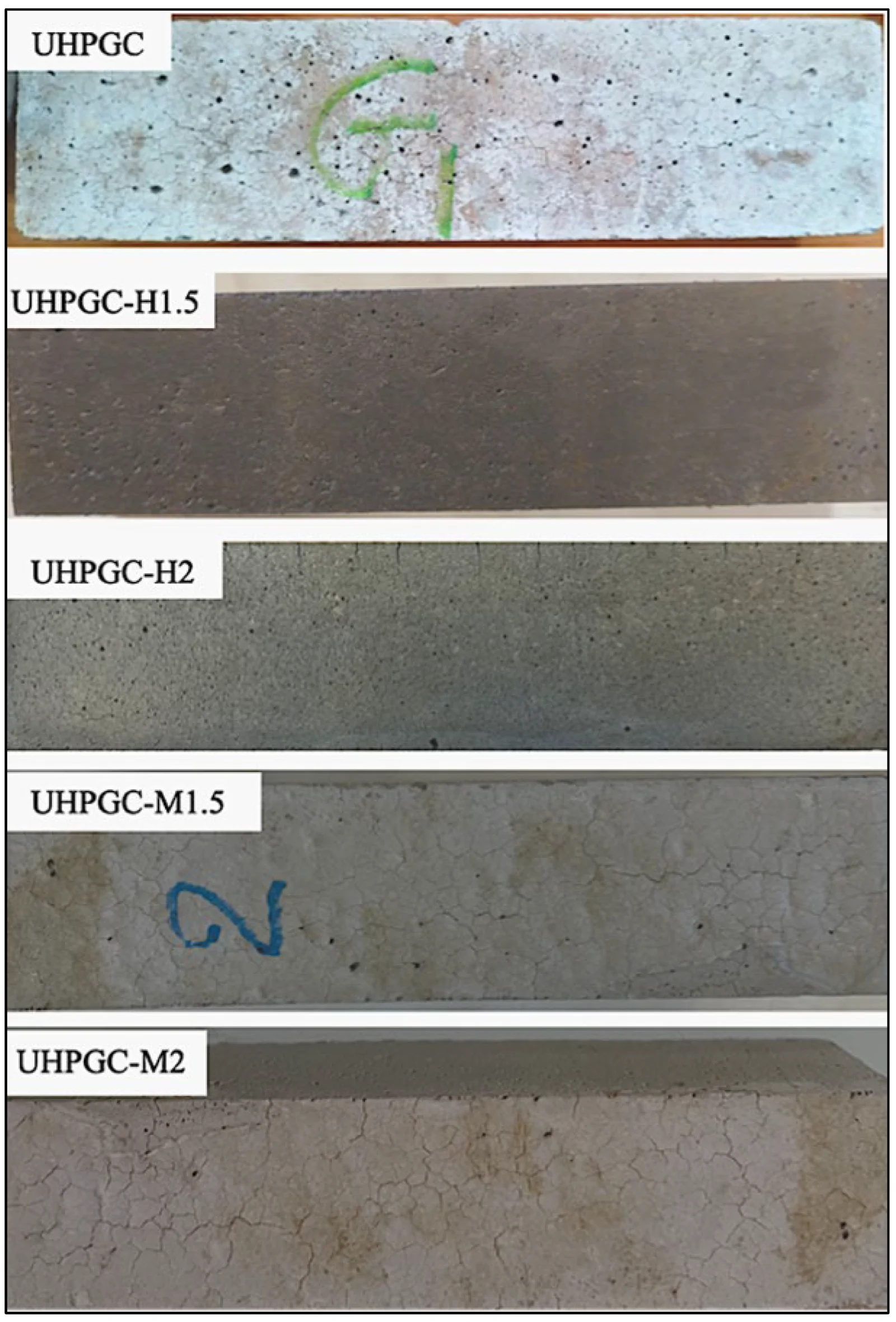
Surface cracks in UHPGC mixtures after exposure to 800 °C.

**Figure 8:**
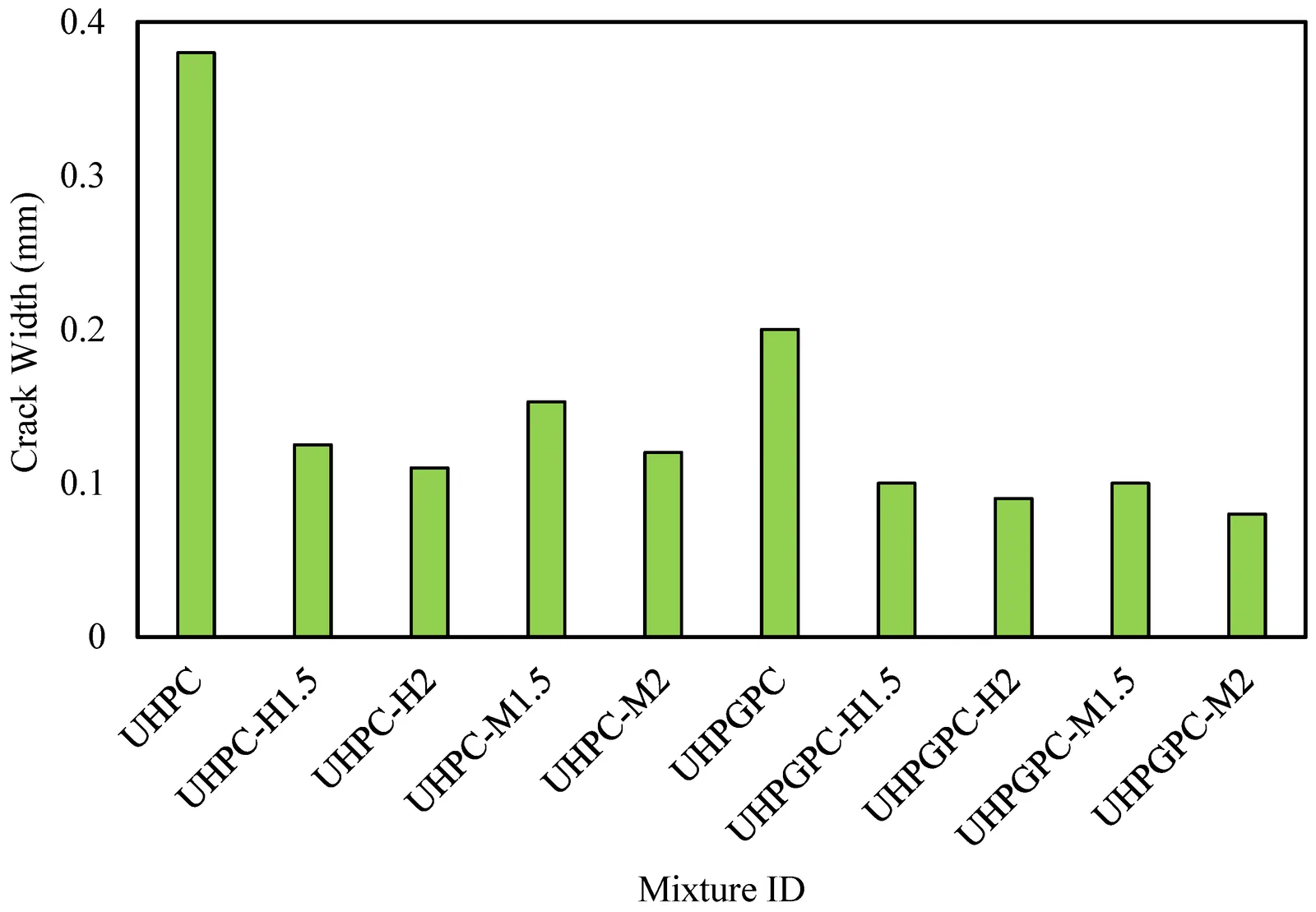
Crack width in the developed specimens after exposure to 800 °C.

Further inspection of the surfaces of the UHPC and UHPGC specimens revealed the development of surface cracks upon exposure to high temperatures. Visual inspections revealed that plain UHPGC specimens developed fewer, less severe surface cracks than UHPC specimens. As shown in Figure 7, the cracks were primarily observed in the UHPGC specimens due to the lack of fibre reinforcement. The whitish surface of the UHPGC specimen is due to the presence of silicate sand. The silicate sand particles were collected and sieved without washing or drying. Steel fibres enhance the concrete’s crack resistance. The observed cracks ranged from fine splits to hairline fractures, as shown in Figure 8. The quantitative results demonstrate that the UHPGC mixtures had 47% higher crack resistance than UHPC (plain). For UHPGC-M2, the finest recorded crack width was 0.08 mm, representing a 79% improvement over plain UHPC.

The primary cause of these cracks is the differential thermal expansion among the aggregate particles, the cement matrix, and the steel fibres. This leads to tensile stresses that exceed the material’s tensile strength, resulting in cracking and potential spalling. Previous studies have shown that exposure of UHPC to elevated temperatures causes microstructural deterioration, increased porosity, weakening of the fibre–matrix interface, and changes in hydration products, which contribute to reduced mechanical performance and the development of thermal cracking [60,61]. However, no explosive spalling was observed in the developed specimens. This outcome is attributed to the 24 h dry-oven pre-treatment at 105 °C, which effectively reduces interstitial moisture before exposure to high temperatures. Previous studies used lower pre-treatment temperatures; however, their specimens still experienced spalling [14]. This study suggests that appropriate curing conditions and pre-treatment protocols substantially reduce the risk of explosive spalling [62,63,64]. Although this protocol is used under laboratory conditions rather than in real fire exposure, it is based on established studies. It can be recommended as a practical pre-treatment method for protecting critical structural members in construction. Thus, it represents a step forward with direct industrial applicability.

After exposure to 800 °C, all the specimens remained solid, with only colour changes and the development of surface cracks. As shown in Figure 5b and Figure 6b, the cross-sections of the specimens after flexural strength testing exhibit sharp, solid edges. This observation suggests that the optimised mix design and proper curing are significant in maintaining concrete’s structural integrity after exposure to high temperatures. It is also noteworthy that the strength performance of the concrete specimens was unaffected by surface cracks.

While all fibres reduced cracking, the 2% micro-steel fibre dosage in UHPGC-M2 proved optimal, achieving the finest crack width of 0.08 mm and outperforming the 1.5% dosage, making it the most viable strategy for resisting thermal cracks. However, if the cracks were wider, it is possible that micro steel fibres would not have held the thermal cracks. Yet, no specimens experienced pronounced structural cracks.

Unlike studies favouring hooked fibres [9,24]. This work demonstrates that microfibres offer superior crack control in dense UHPGC matrices under fire exposure. However, this study further indicates that micro steel fibres provided superior post-exposure crack control relative to hooked-end fibres. This contradicts some of the literature, which reports that hooked-end fibres are the most effective at enhancing toughness [24]. However, a study has also shown that hybrid fibres, including steel fibres, can improve ductility in geopolymer concrete after high-temperature exposure. Nonetheless, this depends on the interaction between the fibres and the concrete matrix. The improved distribution and higher surface area of microfibres within the dense matrix of UHPGC mixtures have resulted in fewer surface cracks. Additionally, although previous studies focused on short-term exposures, the 2 h exposure at 800 °C in this study may have amplified differences in fibre performance.

#### 3.1.3. Chemical Analysis

The chemical composition of raw powders and crushed concrete specimens was measured using X-ray fluorescence. Samples were ground to <75 μm and corrected for loss-on-ignition (LOI). Raw powders were analysed to determine the baseline composition, and post-800 °C specimens were analysed to assess the thermal-induced chemical changes. Table 5 summarises the changes in chemical composition for both concrete types. The percentage losses were calculated using the following equation.

(2) $$Oxides\;Loss\;(\%)=\frac{Initial\;oxide-Final\;oxide}{Initial\;oxide}\times 100$$

In UHPC, a significant loss of the principal oxides was observed after thermal exposure. SiO_2_, the most dominant oxide, decreased by 98.84%, indicating severe degradation of silicate-based phases at elevated temperatures [65,66]. The Al_2_O_3_ content dropped by 95.98%, reflecting the decomposition of aluminosilicate hydrates. CaO, a key component in portlandite and C-S-H gel, decreased by 99.94%. This is attributed to the decomposition of calcium-bearing hydration products and subsequent redistribution into newly formed high-temperature phases.

The UHPGC specimens exhibited a similar degradation trend, although with subtle differences in oxide stability. SiO_2_ was reduced by 99.41% and Al_2_O_3_ by 99.46%. This concentration loss is associated with the restructuring of aluminosilicate networks and changes in phase assemblage during thermal exposure. Additionally, SiO_2_ is a crucial component that aids in the formation of the Si–O–Al network in the Sodium Alumino-Silicate Hydrate (N-A-S-H) gel. This confirms the thermal instability of sulphate-bearing compounds even in the geopolymer system. CaO, although present in lower quantities than UHPC, showed a 99.51% decrease, indicating the decomposition of minor calcium phases within the matrix.

UHPGC-M2 showed the lowest mass loss of 4.8%, yet XRF indicated substantial apparent oxide losses up to 99%. The results indicate decomposition trends rather than complete removal and confirm that UHPGC retained better thermal stability despite chemical redistribution. The superior thermal stability of UHPGC is attributed to its aluminosilicate geopolymer gel (N-A-S-H), which forms a stable three-dimensional Si-O-Al network [11]. Unlike the calcium silicate hydrate (C-S-H) gel in OPC-based UHPC, which progressively dehydrates and decomposes above 300 °C, the N-A-S-H network retains greater structural cohesion at elevated temperatures, thereby limiting microcracking and mass loss. Nevertheless, geopolymer concrete exhibits superior durability and lower porosity than OPC concrete at high temperatures. In the present study, UHPGC showed lower porosity and fewer microstructural changes than UHPC. This observation supports the assertion that UHPGC developed fewer microstructural cracks than OPC. Thus, UHPGC offers greater durability than UHPC.

Chemical analysis before and after thermal exposure showed greater oxide retention in UHPGC than in UHPC. For instance, CaO loss in UHPC reached 99.94%, compared to 99.5% in UHPGC. While these values are close, the higher initial calcium content in UHPC makes the degradation more structurally impactful. This supports prior findings from Phan et al. [11]. However, this study is among the few that directly measure oxide loss post-fire exposure using XRF based on UHPGC. The results suggest that the three-dimensional aluminosilicate gel network in UHPGC provides a more stable framework at elevated temperatures. However, the extreme loss of all principal oxides still indicates marked microstructural changes that could affect long-term durability, a topic beyond the scope of this study.

#### 3.1.4. Mechanical Properties

##### Uniaxial Compressive Strength

The residual mechanical performance of UHPC and UHPGC specimens after high-temperature exposure was evaluated using compressive strength testing, as shown in Table 6. All results are reported as the mean standard deviation (SD) with *n* = 10 specimens per mixture. The compressive strength results highlight apparent differences between UHPC and UHPGC binders, as well as between hooked-end and micro steel fibres, at dosages of 1.5% and 2%. Under ambient conditions, UHPGC consistently outperformed UHPC, with UHPGC-M2 reaching 120.8 MPa compared to 113.5 MPa for UHPC-M2. This shows that geopolymers form a dense N-A-S-H gel matrix that is more thermally stable than the C-S-H gel found in conventional UHPC [67,68,69].

After exposure to 800 °C, both concretes experienced severe degradation, but the extent differed. The plain UHPC specimen retained only 19% of its initial strength (19.6/101.3 MPa), whereas UHPGC retained 24% of its initial strength (20.3/108.2 MPa). Fibre reinforcement considerably improved performance in both systems, with the effect more pronounced in UHPGC. Particularly, when microfibres were used. UHPC-M2 retained 21% of its strength (24/113.5 MPa), while UHPGC-M2 retained 25% (30/120.8 MPa).

The fibre geometry also governed performance. Micro steel fibres provided higher peak strengths under ambient conditions: UHPC-M2 was 113.5 MPa and UHPGC-M2 was 120.8 MPa, respectively, due to better dispersion and crack bridging across fine microcracks. Hooked fibres, by contrast, offered more controlled post-peak behaviour through enhanced pull-out resistance. After fire exposure, the advantage of microfibres in strength retention became clearer, with UHPGC-M2 (30 ± 0.4 MPa) and UHPGC-M1.5 (27.4 ± 0.4 MPa) outperforming UHPGC-H2 (26 ± 0.5 MPa) and UHPGC-H1.5 (24.5 ± 0.7 MPa), respectively.

**Figure 9:**
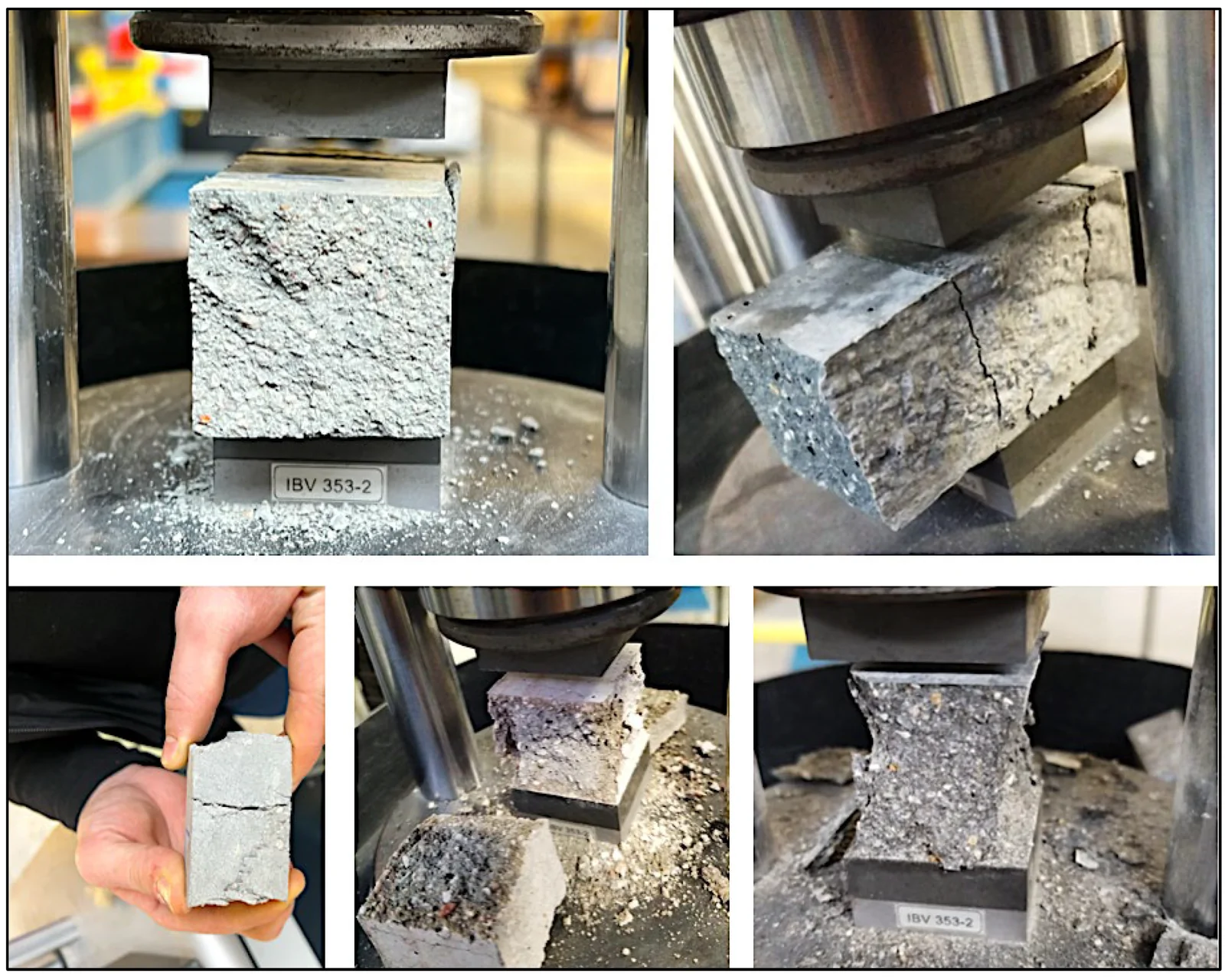
Samples and typical failure shape.

An essential outcome of this study is identifying an efficiency threshold between 1.5% 2% fibre dosage. Across both binders, strength improvements beyond 1.5% were modest, as seen in UHPC-M1.5 (23 ± 0.6 MPa) and UHPC-M2 (24 ± 0.3 MPa), indicating that the differences are within experimental variability. Hence, this suggests diminishing returns at doses above this level. Other studies have also shown that an excessive fibre content reduces workability and limits further strength gains [70]. Thus, a fibre content of 1.5–2% can be considered optimal for post-fire compressive performance, as it balances residual strength and material economy. Figure 9 demonstrates samples and their typical failure shape.

**Figure 10:**
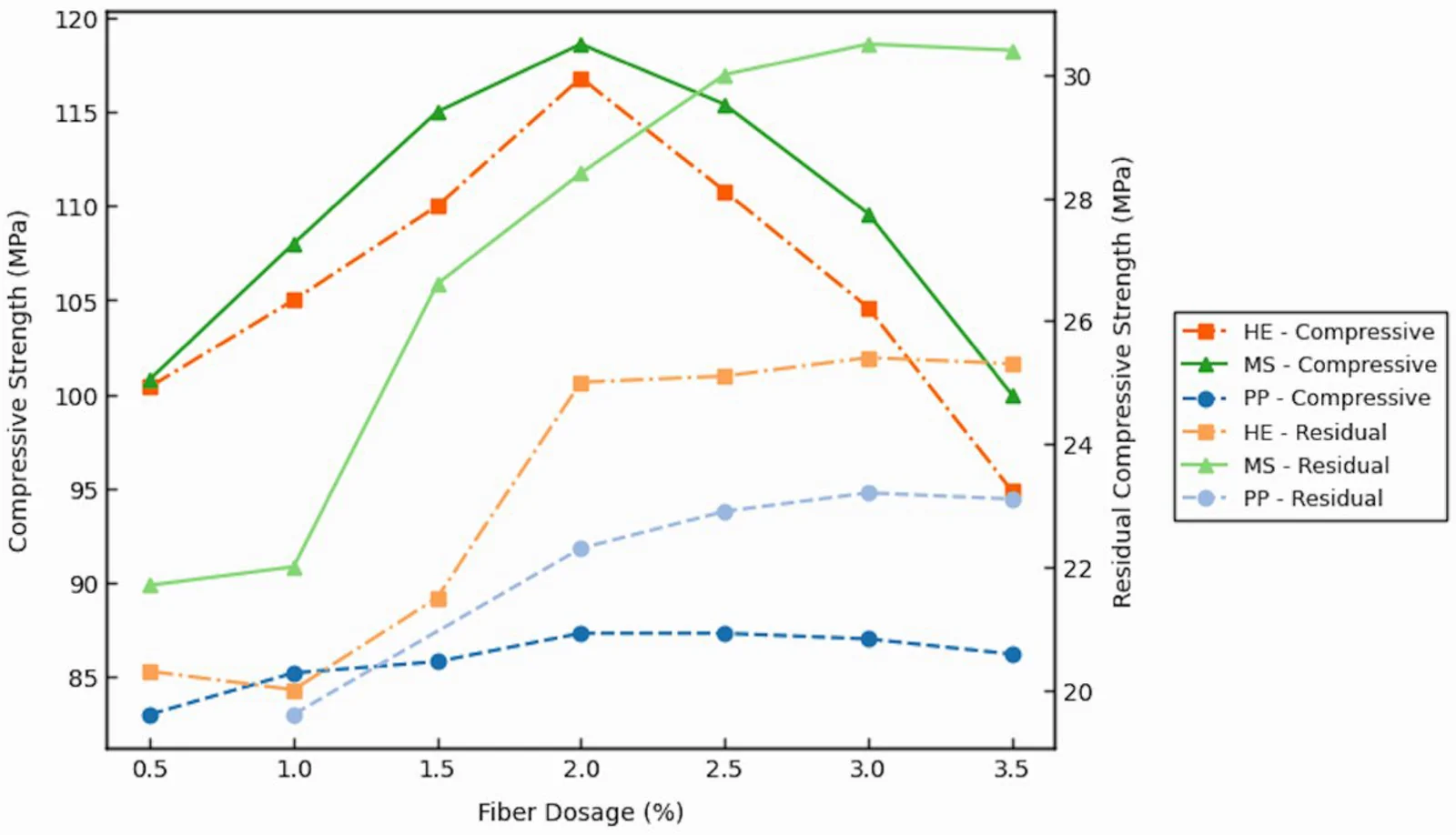
Variation of compressive strength with fibre type and dosage (trial results before mix design optimisation).

This observation was reinforced by a detailed evaluation of dispersion and efficient reinforcement, as shown in Figure 10. The optimum fibre dosage for UHPGC was found to be 1.5–2% for both steel fibres. Thus, this range effectively balances strength enhancement and workability. This is likely due to the distribution of the fibres and crack bridging, which contributed to enhanced overall and residual compressive strength. This is due to clustering, porosity, and internal defects. The MS fibres provide superior crack control and retention, while HE fibres enhance peak compressive resistance through mechanical anchorage. For polypropylene (PP) fibres, improvements in compressive and residual strength were minimal, indicating that their primary role is to enhance spalling resistance rather than strengthen the material.

These findings align with previously reported strength-retention ratios ranging from 15% to 85% for high-temperature-exposed concrete. Still, this study extends prior work by directly comparing UHPC and UHPGC under identical fire conditions and by quantifying the efficiency zone of fibre reinforcement. The ratios obtained in this study fall at the higher end due to the optimised fibre content. One limitation is that no microstructural imaging was conducted to validate pore evolution, which could strengthen these conclusions in future work.

##### Flexural Strength

**Figure 11:**
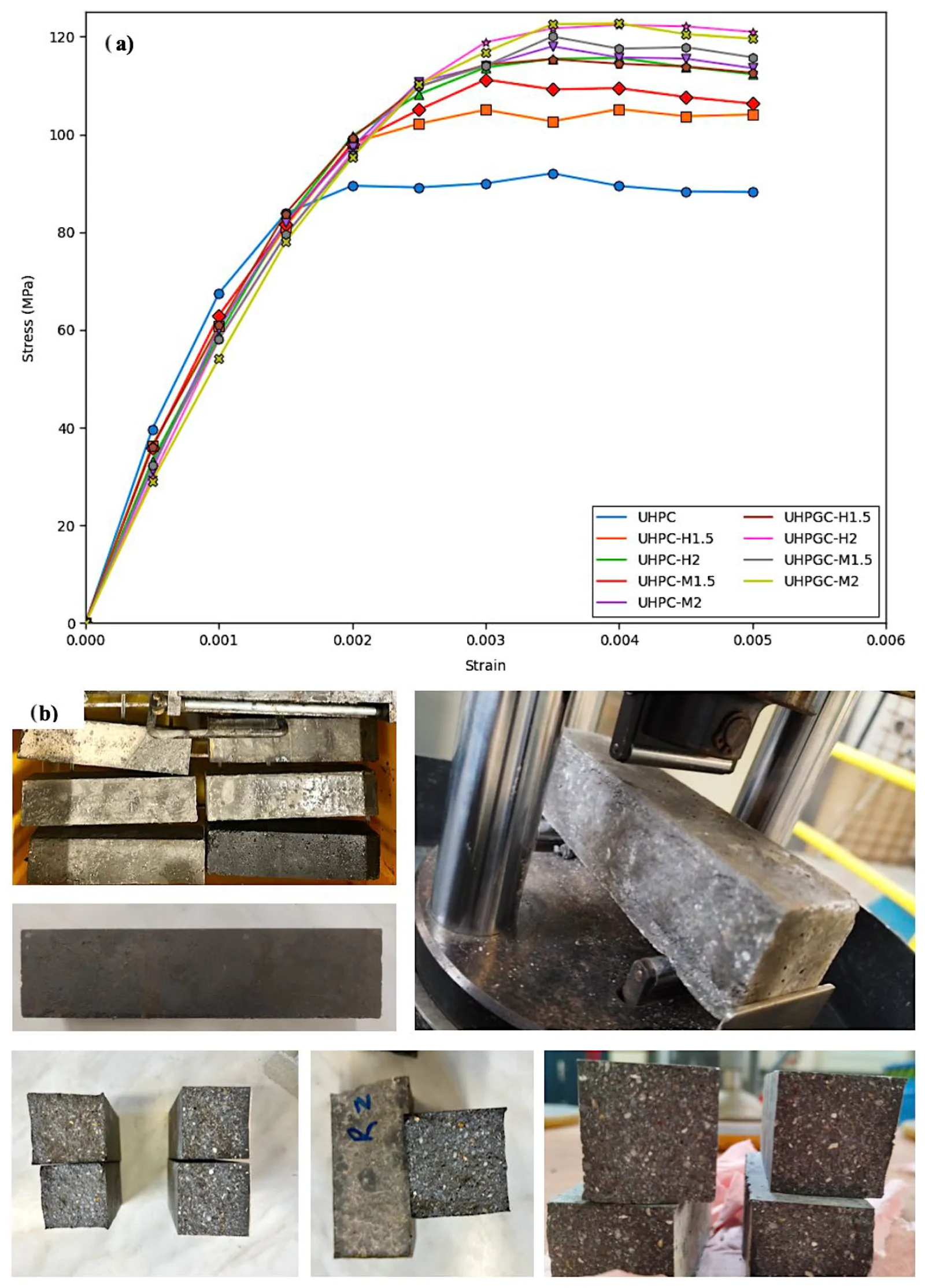
(**a**) Maximum load–deflection curves for UHPC and UHPGC specimens under room temperature conditions, and (**b**) samples and typical shape failure.

**Figure 12:**
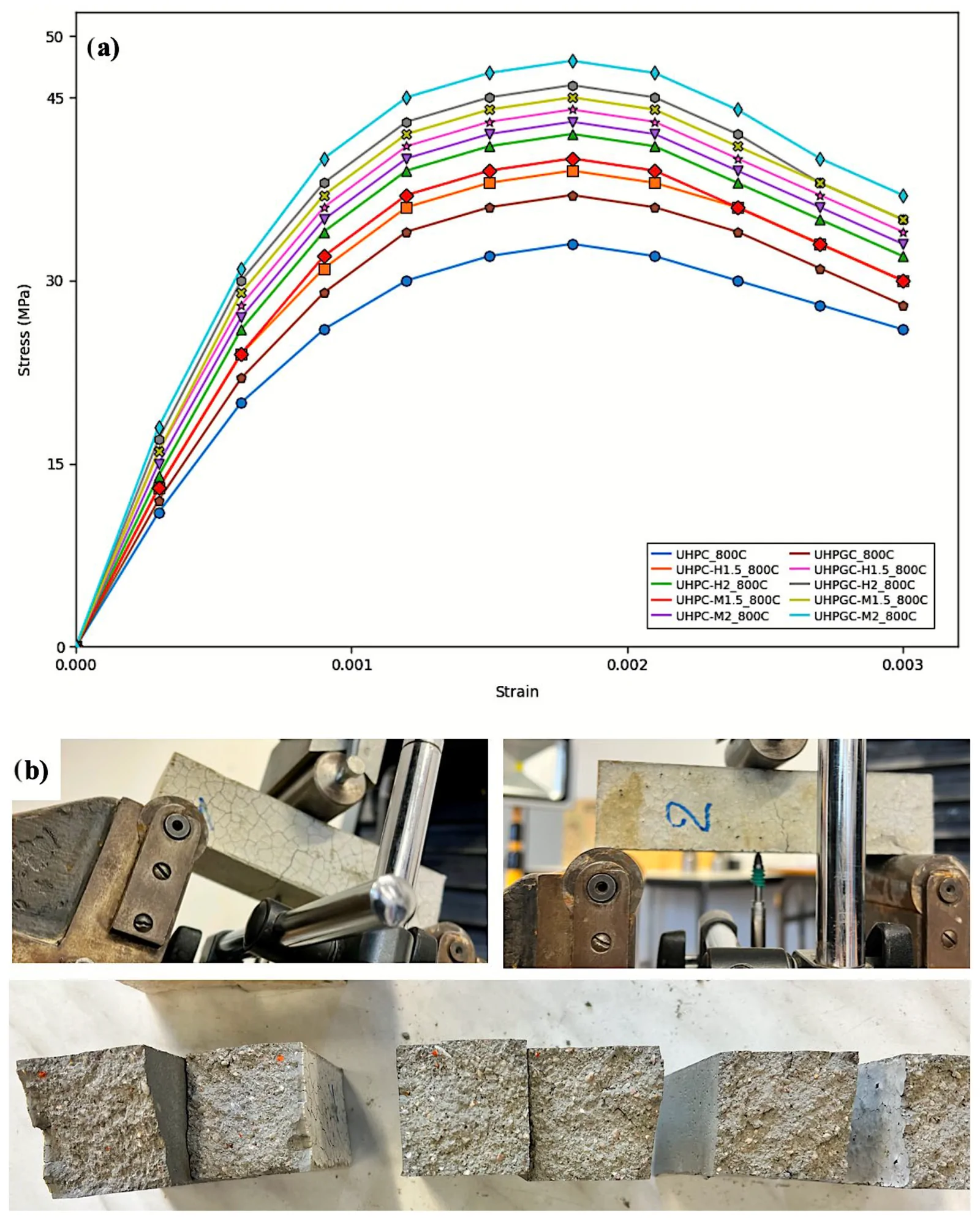
(**a**) Maximum load–deflection curves for UHPC and UHPGC specimens after exposure to 800 °C, and (**b**) samples and typical failure shape.

The maximum load–deflection curves for UHPGC and UHPC mixtures exhibited different performance trends under ambient and high-temperature exposure conditions, as illustrated in Figure 11 and Figure 12. At room temperature, all mixtures exhibited nonlinear ascending load–deflection behaviour, followed by post-peak softening. Hooked-end steel fibres at 2% improved post-peak ductility and deflection capacity, due to enhanced fibre anchorage and crack-bridging efficiency. In contrast, micro steel fibres exhibited higher peak flexural strength, as their finer geometry and greater surface area facilitated stress transfer across microcracks, resulting in greater strain capacity but an earlier post-peak stress decline.

After exposure to 800 °C, all specimens exhibited plastic deformation, a reduction in peak stress, and a diminished strain capacity, primarily due to microcracking, fibre debonding, and partial melting. Nevertheless, hooked-end fibre-reinforced specimens retained a more stable descending branch, highlighting their superior post-fire ductility.

UHPGC-M2 reached the highest peak flexural strength at 28 MPa under ambient conditions and maintained the highest residual flexural strength after fire exposure. Microfibres effectively minimised thermally induced microcracks at the matrix level, while hooked fibres improved post-peak load transfer and controlled failure. The residual flexural strength retention across all mixtures was up to 20%. This demonstrates significant deterioration in the flexural strength and fracture performance of UHPC concrete after exposure to temperatures up to 800 °C [66]. However, this started from a higher baseline due to the thermally resilient geopolymer binder and the optimised fibre distribution.

**Figure 13:**
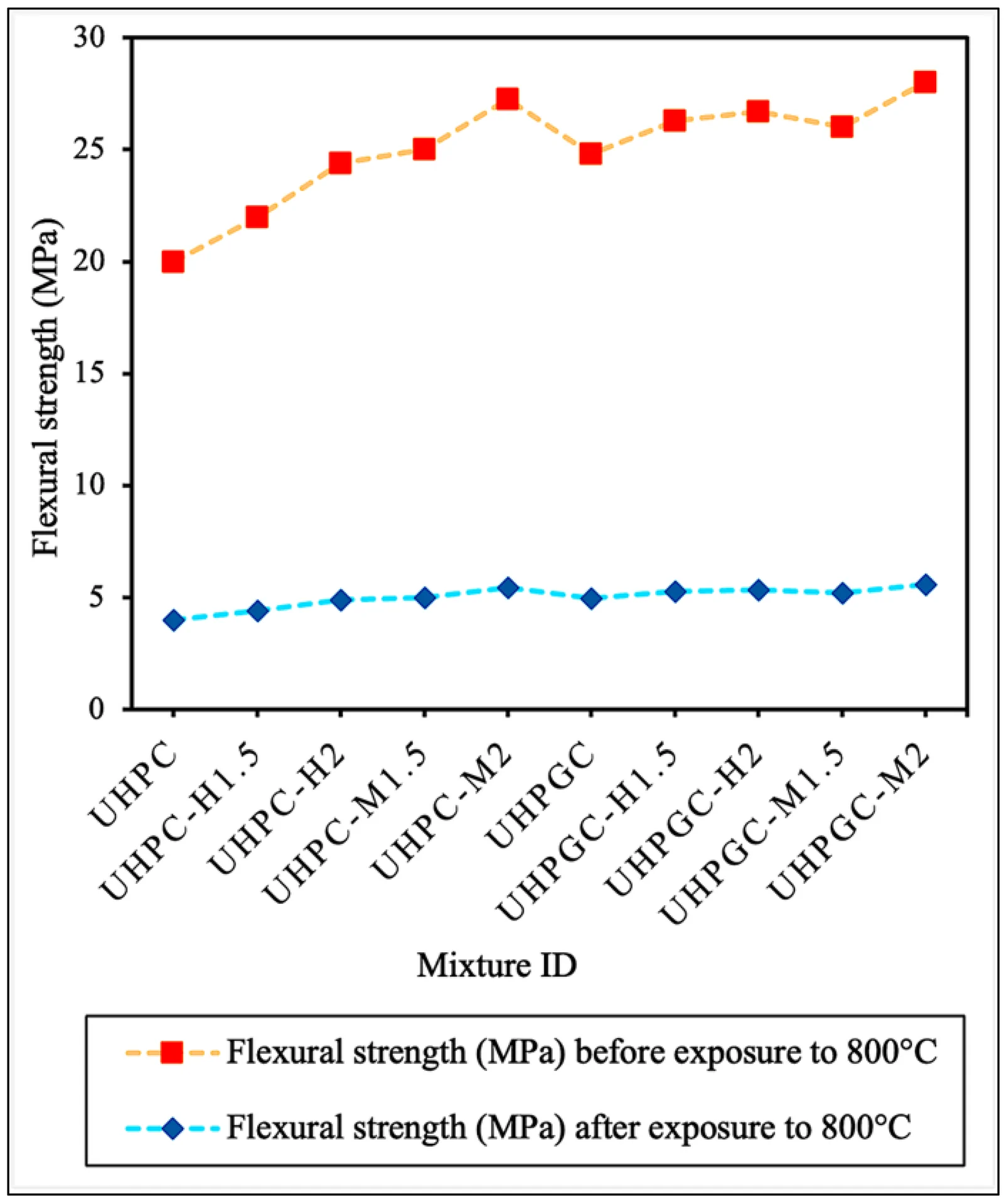
The maximum flexural strength results before and after exposure to 800 °C.

The flexural behaviour of UHPGC and UHPC mixtures differed markedly with fibre type and dosage, as illustrated in Figure 13. Under ambient conditions, micro steel fibres at a 2% dosage resulted in higher peak flexural strength due to their ability to facilitate heat transfer across microcracks. UHPGC-M2 reached 28 MPa, the highest among all mixtures. Nonetheless, hooked-end fibres provided superior post-cracking ductility. The superior anchorage within the concrete matrix enabled gradual load transfer after peak load. This results in a stable descending branch in the load–deflection curve.

Hence, their interaction with the concrete matrix explains the differences between fibre types. Hooked-end fibres improve post-peak behaviour through mechanical anchorage, delaying crack propagation, whereas microfibres increase peak strength through uniform dispersion and bridging fine microcracks. UHPGC’s superior performance compared to UHPC arises from the stable aluminosilicate (N-A-S-H) gel network, which better redistributes stress and limits microcracking under thermal loads [11]. The flexural failure modes corroborate these trends. Room-temperature specimens exhibited uniform failure planes due to dense matrix packing and fibre reinforcement. The flexural failure modes corroborate these trends. Room-temperature specimens exhibited uniform failure planes due to dense matrix packing and fibre reinforcement. In contrast, specimens exposed to 800 °C exhibited irregular, fragmented failure planes resulting from matrix dehydration and decomposition.

##### Splitting Tensile Strength

**Figure 14:**
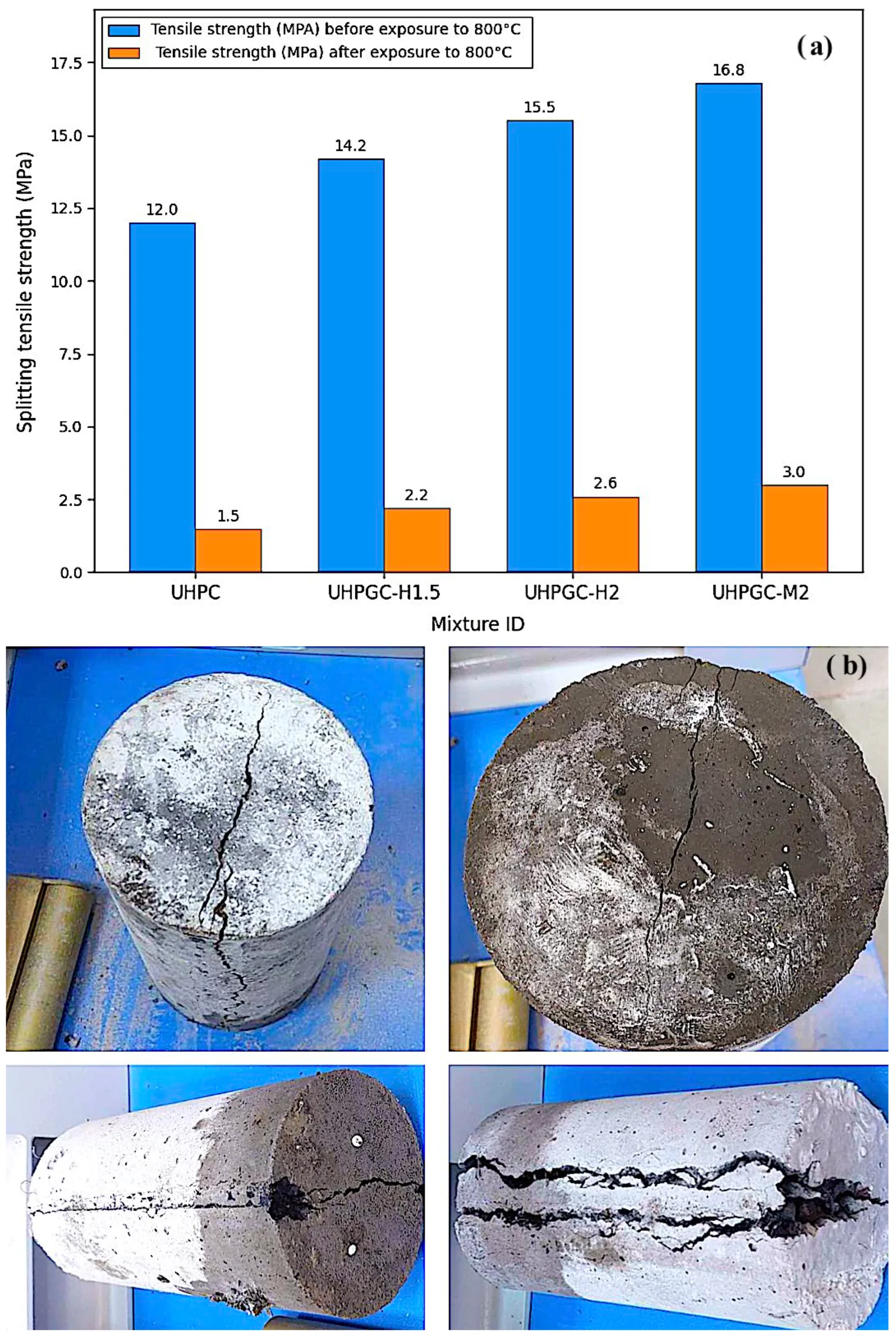
(**a**) The maximum tensile strength before and after exposure to 800 °C, and (**b**) the sample and shaping failure.

The splitting tensile strengths of UHPC and UHPGC mixtures were relatively close under ambient conditions, as shown in Figure 14a,b. The splitting tensile strength of the cylindrical specimens was calculated using Equation (3):

(3) $$T\;=\frac{2P}{\pi ld}$$

where *T* is the splitting tensile strength (MPa), *P* is the maximum applied load at failure (N), *l* is the length of the cylindrical specimen (mm), and *d* is the diameter of the cylindrical specimen (mm).

Only slight variations among mix designs indicated that compositional changes had a moderate effect on tensile performance. At room temperature, UHPGC-M2 achieved the highest tensile strength of 16.8 MPa, whereas plain UHPC exhibited the lowest value of 12 MPa. The incorporation of hooked-end and micro steel fibres noticeably enhanced the tensile capacity by providing effective crack-bridging mechanisms and delaying crack propagation.

After exposure to 800 °C, all mixtures experienced substantial reductions in tensile strength, with residual values ranging from 1.5 MPa for UHPC to 3 MPa for UHPGC-M2. This corresponds to a strength loss of up to 88%, confirming that tensile performance is the most vulnerable property under extreme thermal exposure. Despite this severe reduction, UHPGC mixtures consistently outperformed UHPC, achieving higher residual tensile strength due to the geopolymer binder’s thermal stability. The dense, cross-linked N-A-S-H gel structure in UHPGC helps preserve matrix cohesion and mitigate crack propagation at high temperatures. In contrast, UHPC relies on a C-S-H gel, which begins to decompose at temperatures between 300 and 600 °C [11,17].

Additionally, high pore pressures from water evaporation contribute to internal damage and spalling in UHPC, thereby accelerating strength loss [70]. Notably, UHPGC exhibited slightly higher tensile retention, highlighting the aluminosilicate matrix’s role in maintaining the load-bearing capacity under extreme heat conditions. Hence, changes in the fibre type and dosage had a greater influence on the post-cracking response and fracture energy than on the peak tensile strength of UHPC [70,71,72,73].

While UHPGC exhibited better retention than UHPC, the overall loss underscores that tensile strength remains highly susceptible to thermal degradation. These results also reveal a gap in the literature regarding the optimisation of post-fire tensile performance. Future studies should investigate strategies such as hybrid fibre systems, higher fibre dosages, or nano-additives to enhance residual tensile capacity, potentially mitigating the extreme losses observed at high temperatures.

### 3.2. Main Factors Influencing UHPC and UHPGC Performance

#### 3.2.1. Fibre Reinforcement

Fibre characteristics, including the type, geometry, aspect ratio, and dosage, play a decisive role in the residual mechanical performance of UHPC and UHPGC after thermal exposure. However, the relationship is not linear. Several studies have identified an optimal fibre dosage beyond which matrix homogeneity deteriorates due to fibre agglomeration, resulting in a weaker interfacial transition zone (ITZ) and diminished mechanical performance rather than further improvement [26,34]. High-aspect-ratio and micro-scale steel fibres generally provide superior post-fire performance compared with conventional hooked-end fibres because they promote more uniform crack bridging and delay crack coalescence at elevated temperatures [27,37]. At intermediate temperatures (≤400 °C), temporary increases in compressive strength have occasionally been reported. These gains are primarily attributed to continued hydration and pore refinement rather than to fibre reinforcement itself [35].

#### 3.2.2. Binder System

The binder composition is consistently identified as the dominant factor governing high-temperature resistance. Geopolymer binders based on GGBS and fly ash activated with NaOH/Na_2_SiO_3_ generally exhibit greater thermal stability than OPC-based UHPC, retaining a higher proportion of both compressive and flexural strength following equivalent thermal exposure [28,34]. This superior performance is commonly associated with the higher thermal stability of aluminosilicate gel networks and the absence of portlandite decomposition. Optimisation of the alkaline activator modulus, together with one-part geopolymer formulations, has been shown to enhance residual mechanical properties further when combined with an appropriate fibre system [34]. For OPC-based UHPC, an increased silica fume content and hybrid multi-scale fibre reinforcement (e.g., steel–PVA) can substantially improve post-fire performance. However, these measures reduce rather than eliminate the performance gap relative to geopolymer systems [26,30].

#### 3.2.3. Aggregate Characteristics

Aggregate selection also influences thermal behaviour by affecting stress redistribution and thermal compatibility within the matrix. Moderate coarse aggregate contents (approximately 15%) have been reported to improve load transfer and peak load capacity. In contrast, higher replacement levels (approximately 30%) tend to increase thermal incompatibility within the ITZ and accelerate deterioration during heating [26]. Properly graded recycled coarse aggregates have demonstrated thermal performance comparable to that of natural aggregates [32]. In contrast, mortar-based formulations without coarse aggregates generally exhibit lower thermal mass loss because the reduced thermal expansion mismatch limits the development of internal stresses [27].

#### 3.2.4. Thermal Exposure Conditions

Material degradation does not progress linearly with increasing temperature. Exposure to 300–400 °C may produce modest strength gains owing to continued hydration and matrix densification. Between 600 and 700 °C, the dehydration of hydration products becomes dominant, leading to significant reductions in mechanical properties, although well-designed fibre-reinforced systems frequently retain 70–90% of their original strength. Above 800 °C, extensive microcracking, matrix decomposition, and bond deterioration lead to severe strength loss, with mass losses typically remaining at 7–10% despite substantially greater reductions in mechanical performance [27,33,37]. At temperatures approaching or exceeding 1000 °C, degradation of the binder phase becomes critical, and only hybrid systems incorporating vapour-release fibres, such as polypropylene, retain limited structural integrity. In addition, specimen conditioning before testing affects the measured performance. Extended air-drying has been shown to reduce apparent thermal mass loss compared with conventional 24 h oven drying by lowering the initial moisture content [41].

### 3.3. Crack Propagation and Visual Deterioration

Thermal exposure substantially accelerates crack initiation and propagation in both UHPC and UHPGC. Based on the reviewed studies, thermal deterioration becomes more pronounced at approximately 500 °C and intensifies significantly at 800 °C, where severe crack propagation is consistently observed [51]. Reported crack widths typically increase from approximately 0.05 mm before heating to 0.25–0.45 mm after exposure to 800 °C, although the extent of cracking depends strongly on the fibre characteristics. Optimised fibre dosages can reduce crack widths by up to fivefold compared with unreinforced specimens by effectively bridging cracks. In contrast, excessive fibre contents promote clustering and local stress concentrations that accelerate crack development [34]. The fibre geometry and binder chemistry further influence crack resistance, with potassium-activated geopolymer systems generally exhibiting the smallest crack widths and the highest residual splitting tensile strength among the formulations investigated [36]. Hence, geopolymer concrete demonstrates greater resistance to thermally induced crack propagation than OPC-based ultra-high-performance concrete, particularly when reinforced with an optimum fibre dosage.

Visual changes in the specimen colour provide useful qualitative indicators of the underlying degradation mechanisms. OPC-based UHPC commonly changes from grey to brownish-white upon heating, reflecting the dehydration of the C–S–H gel and the decomposition of portlandite. In contrast, geopolymer systems generally develop brick-red or brown hues associated with iron oxide formation during decomposition of the aluminosilicate gel network [28,34]. Importantly, the relatively small mass losses typically recorded (<10%) stand in sharp contrast to the much larger reductions in mechanical properties, which frequently exceed 60%. This disparity indicates that deterioration is governed primarily by microstructural damage, phase transformations, and crack development rather than by bulk material loss alone.

### 3.4. Comparison with Previous Studies

Direct comparisons between UHPC and UHPGC subjected to identical thermal exposure remain limited in the literature. Nevertheless, available studies consistently demonstrate that geopolymer binders provide superior residual compressive strength compared with OPC-based systems following exposure to approximately 800 °C. For example, geopolymer concretes activated with either sodium or potassium retained approximately 60% of their original compressive strength, whereas comparable OPC concretes retained only around 40% under identical heating conditions [36]. This advantage progressively diminishes at temperatures approaching 900 °C, where all binder systems converge toward similarly low residual strengths, suggesting that both C–S–H and aluminosilicate gel networks eventually reach comparable thermal degradation limits [39]. Compared with the influence of binder chemistry, fibre reinforcement and aggregate modification generally provide secondary improvements of approximately 10–15% in residual performance [28].

The findings of the present study align well with this established trend. UHPGC-M2 retained 25% of its original compressive strength after exposure to 800 °C (120.8–30 MPa), compared with 21% for UHPC-M2 (113.5–24 MPa), confirming the superior thermal stability of the geopolymer matrix. Although the percentage retention observed here is lower than that reported in some previous investigations, the residual compressive strength achieved by UHPGC-M2 is among the highest reported for geopolymer concrete exposed to this temperature. This outcome reflects the exceptionally high initial strength of the developed mix and highlights the importance of considering both absolute residual strength and percentage retention when evaluating thermal performance.

This distinction becomes evident when comparing the present results with previous studies. Shaikh and Hosan [36] reported 60% compressive strength retention in sodium-activated geopolymer concrete (60–36 MPa), compared with 40% in OPC concrete (55–22 MPa). Similarly, Wang et al. [28] observed 49–51% retention for geopolymer concrete after exposure to 700 °C (68–78–39–41 MPa). Although these retention percentages exceed those obtained in the present study, their residual strengths remain considerably lower because the initial compressive strengths were substantially lower. Consequently, the lower percentage retention reported here primarily reflects the greater thermal severity (800 °C) and the much higher initial strength of the developed UHPGC rather than inferior material performance. Likewise, Amin et al. [32] achieved approximately 38% retention in hybrid steel–carbon fibre UHPC (188–72 MPa), illustrating the beneficial contribution of hybrid fibre reinforcement within OPC systems while remaining consistent with the overall superiority of geopolymer binders.

The flexural and tensile responses observed in the present study are similarly consistent with published trends. Kong et al. [37] reported flexural strength reductions of approximately 22–26% following exposure to 600 °C, whereas the larger reductions measured at 800 °C in the present work are consistent with the greater degree of thermal damage expected at higher temperatures. For splitting tensile strength, UHPGC-M2 retained 17.9% of its initial capacity (16.8–3.0 MPa), comparable to the retention reported by Zhang et al. [34], despite beginning from a substantially higher initial strength. These comparisons further demonstrate that percentage retention alone does not fully capture post-fire structural performance.

Crack development also distinguishes the present formulations from those previously reported. The maximum post-fire crack width measured in UHPGC-M2 was only 0.08 mm, considerably lower than the 0.25–0.40 mm range reported by Zhang et al. [34] and Wang et al. [28]. This improved crack resistance is attributed to the use of finer steel fibres, which enhanced crack bridging and limited crack propagation throughout the geopolymer matrix. Visual deterioration followed the degradation mechanisms consistently reported in the literature, with OPC-based UHPC exhibiting light grey or whitish discolouration associated with C–S–H dehydration, while UHPGC developed grey-brown to orange colouration linked to iron oxide formation during aluminosilicate decomposition [28,34].

## 4. Conclusions

Based on the results of this experimental investigation on UHPC and UHPGC, examining the effect of steel fibres at elevated temperatures and supported by an extensive literature database, the following can be concluded:

- Chemical analysis indicated substantial chemical redistribution in both systems after exposure to 800 °C; however, UHPGC exhibited lower mass loss and higher residual strength than OPC-based UHPC. This behaviour may be associated with the greater thermal stability of the N-A-S-H geopolymer network relative to the C-S-H gel in OPC systems, as widely reported in previous studies.
- Although both materials experienced substantial strength loss after exposure to 800 °C, UHPGC consistently outperformed UHPC. The UHPGC mix reinforced with 2% micro steel fibres (UHPGC-M2) achieved the highest residual compressive strength of approximately 30 ± 0.4 MPa, with approximately 25% retention, compared to about 24 MPa for the corresponding UHPC mix.
- Fibre geometry played a critical role in post-fire performance. Micro steel fibres were more effective in enhancing peak load capacity and limiting micro-crack propagation due to their higher specific surface area and improved crack-bridging efficiency. In contrast, hooked-end fibres primarily contributed to post-peak ductility and energy absorption through mechanical anchorage.
- Splitting tensile strength was identified as the most temperature-sensitive mechanical property, with reductions of up to 88% at 800 °C. Nevertheless, fibre reinforcement effectively prevented complete disintegration, with micro steel fibres in the UHPGC matrix providing the highest relative retention of tensile capacity.
- Visual inspection confirmed that geopolymer binders substantially mitigated thermal cracking. UHPGC specimens exhibited fewer and narrower cracks than UHPC, with the UHPGC-M2 mix recording the minimum crack width of approximately 0.08 mm, indicating superior restraint of thermal expansion and internal vapour pressure.
- Colourimetric changes revealed distinct degradation mechanisms. UHPC specimens transitioned to a light grey or white colour due to calcium-based dehydration reactions. In contrast, UHPGC specimens exhibited an orange-brown discolouration associated with iron oxidation within the aluminosilicate framework, further evidencing the different thermal responses of the two binder systems.
- A fibre content between 1.5% and 2.0% was identified as the effective threshold for ultra-high-performance composites exposed to fire. Dosages below this range compromised thermal resistance, while higher contents resulted in diminished mechanical benefits and reduced workability, as observed during the trial stage. The 2.0% micro steel fibre dosage provided the optimal balance among workability, density, and residual strength.
- The findings of this study demonstrate that UHPGC reinforced with micro steel fibres offers superior residual mechanical performance and thermal stability after fire exposure compared with OPC-based UHPC. These results support its potential application in fire-resistant structural elements, tunnels, high-rise buildings, industrial facilities, and other infrastructure that require enhanced post-fire structural integrity.

## Figures and Tables

**Table 1:** The chemical and physical composition of the OPC, GGBS, and fly ash (%).

Material	SiO_2_ (%)	Al_2_O_3_ (%)	Fe_2_O_3_ (%)	CaO (%)	MgO (%)	SO_3_ (%)	Bulk Density (kg/m^3^)	Specific/Apparent Gravity (g/cm^3^)	Fineness (m^2^/kg)	LOI
OPC	23.4	4.8	3	66.5	1.5–2.5	2.0–2.3	1440 ± 0.1	3.10–3.18	320–335	2.2–3.2
GGBS	35.2	9	5.8	45	5	–	1150	2.95	350–450	1–3
Fly ash	55	26	7.3	9.2	2.45	0.23	700–900	3.27	–	0.009

**Table 2:** Properties of steel fibres (provided by the manufacturer).

Steel Fibre Shapes	Diameter (mm)	Tensile (MPa)	Aspect Ratio (L/D)
Micro	0.25	≥2850	56
Hooked end	0.75	>1150	46

**Table 3:** Formulated mix designs (kg/m^3^).

Group	Mixtures	Cement	GGBS	FA	Sand	Water	Superplasticizer	Alkaline Liquid	Fibre	Steel Fibre Type
G1	UHPC	990	-	-	1202	180	28	-	0	-
	UHPC-H1.5	990	-	-	1202	180	28	-	14.85	Hooked-end
	UHPC-H2	990	-	-	1202	180	28	-	19.8	Hooked-end
	UHPC-M1.5	990	-	-	1202	180	28	-	14.85	Micro
	UHPC-M2	990	-	-	1202	180	28	-	19.8	Micro
G2	UHPGC	-	594	396	1202	80	28	240	0	-
	UHPGC-H1.5	-	594	396	1202	80	28	240	14.85	Hooked-end
	UHPGC-H2	-	594	396	1202	80	28	240	19.8	Hooked-end
	UHPGC-M1.5	-	594	396	1202	80	28	240	14.85	Micro
	UHPGC-M2	-	594	396	1202	80	28	240	19.8	Micro
w/c = 0.18										
Alkaline activator solution (AAS)/binder = 0.24										

**Table 4:** Experimental and adjusted mass loss of UHPC and UHPGC mixtures after exposure to 800 °C.

Group	Mixtures	Experimental Mass Loss (%)	LOI Contribution (%)	Fineness Correction (%)	Thermal Mass Loss (%)
G1	UHPC	6.2	1.11	0.27	4.82
	UHPC-H1.5	5.1	1.11	0.27	3.72
	UHPC-H2	3	1.11	0.27	1.62
	UHPC-M1.5	5	1.11	0.27	3.62
	UHPC-M2	4.8	1.11	0.27	3.42
G2	UHPGC	6.4	0.47	0.25	5.68
	UHPGC-H1.5	5.3	0.47	0.25	4.58
	UHPGC-H2	5	0.47	0.25	4.28
	UHPGC-M1.5	5.3	0.47	0.25	4.58
	UHPGC-M2	4.8	0.47	0.25	4.08

**Table 5:** The alterations of oxides in UHPC and UHPGC before and after exposure to 800 °C.

Oxide	Initial (%)	Post-800 °C (%)	% Reduction	Initial (%)	Post-800 °C (%)	% Reduction
	UHPC	UHPC	UHPC	UHPGC	UHPGC	UHPGC
SiO_2_	23.4	0.271	98.84	45.1	0.268	99.41
Al_2_O_3_	4.8	0.193	95.98	17.5	0.0948	99.46
Fe_2_O_3_	3	0.0037	99.88	6.55	0.0039	99.94
CaO	66.5	0.0405	99.94	27.1	0.133	99.51
MgO	2.3	0.5	78.26	3.72	0.0268	99.28

**Table 6:** Compressive and residual strength results.

Mixture ID	Compressive Strength (MPa)	Residual Compressive Strength (MPa)
UHPC	101.3 ± 2	19.6 ± 0.4
UHPC-H1.5	104.8 ± 2.6	20.3 ± 0.3
UHPC-H2	107.5 ± 1.9	21.4 ± 0.4
UHPC-M1.5	110 ± 3.4	23 ± 0.6
UHPC-M2	113.5 ± 2.1	24 ± 0.3
UHPGC	108.2 ± 1.6	20.3 ± 0.5
UHPGC-H1.5	113 ± 2	24.5 ± 0.7
UHPGC-H2	118 ± 2.1	26 ± 0.5
UHPGC-M1.5	120 ± 3.3	27.4 ± 0.4
UHPGC-M2	120.8 ±2.6	30 ± 0.4

## Data Availability

The original contributions presented in the study are included in the article. Further inquiries can be directed to the corresponding author.

## Appendix A

**Table A1:** Investigating the UHPC and UHPGC variables in previous studies.

Reference	Group Name	Concrete Type	Fibre Material	Fibre Type	Fibre Diameter (mm)	Fibre Tensile Strength (MPa)	Fibre Aspect Ratio (L/D)	Fibre Percentage	Aggregate Type	Maximum Aggregate Size (mm)	Binder Type	$T_{pre}$	*T*	$T_{l}$	$C_{a}$	${MC}_{b}$	${MC}_{a}$	$f_{m}$	${f\prime }_{m}$	$f_{c}$	${f\prime }_{c}$	$f_{t}$	${f\prime }_{t}$
[26]	S0CA0-Tk	UHPC	N/A	N/A	N/A	N/A	N/A	N/A	Quartz Sand	0.6	OPC+SF+FA	20	800	0.56–18	Brownish-White	N/A	N/A	5	1.3	N/A	N/A	N/A	N/A
	S1CA0-Tk	UHPC	Copper	Straight	0.22	1000	80	1.200%	Quartz Sand	0.6	OPC+SF+FA	20	800	0.56–18	Brownish-White	N/A	N/A	9.7	2.5	N/A	N/A	N/A	N/A
	S2CA0-Tk	UHPC	Copper	Straight	0.22	1000	80	2.200%	Quartz Sand	0.6	OPC+SF+FA	20	800	0.56–18	Brownish-White	N/A	N/A	14.2	3.6	N/A	N/A	N/A	N/A
	S2CA15-Tk	UHPC	Copper	Straight	0.22	1000	80	2.200%	Natural + Basalt	10	OPC+SF+FA	20	800	0.56–18	Brownish-White	N/A	N/A	25.8	6.5	N/A	N/A	N/A	N/A
	S2CA30-Tk	UHPC	Copper	Straight	0.22	1000	80	2.200%	Natural + Basalt	10	OPC+SF+FA	20	800	0.56–18	Brownish-White	N/A	N/A	20.8	5.3	N/A	N/A	N/A	N/A
[27]	UC-0-20	UHPC	N/A	N/A	N/A	N/A	N/A	0.000%	Sand	2	OPC+FA	200	20	N/A	Gray-White	N/A	N/A	N/A	N/A	199.2	N/A	N/A	N/A
	UC-1-20	UHPC	Steel	Micro	0.12	2830	50	1.000%	Sand	2	OPC+FA	200	20	N/A	Gray-White	N/A	N/A	N/A	N/A	203.7	N/A	N/A	N/A
	UC-2-20	UHPC	Steel	Micro	0.12	2830	50	2.000%	Sand	2	OPC+FA	200	20	N/A	Gray-White	N/A	N/A	N/A	N/A	208.2	N/A	N/A	N/A
	UC-3-20	UHPC	Steel	Micro	0.12	2830	50	3.000%	Sand	2	OPC+FA	200	20	N/A	Gray-White	N/A	N/A	N/A	N/A	213	N/A	N/A	N/A
	UC-6-20	UHPC	Steel	Micro	0.12	2830	50	6.000%	Sand	2	OPC+FA	200	20	N/A	Gray-White	N/A	N/A	N/A	N/A	226.3	N/A	N/A	N/A
	UC-9-20	UHPC	Steel	Micro	0.12	2830	50	9.000%	Sand	2	OPC+FA	200	20	N/A	Gray-White	N/A	N/A	N/A	N/A	239.9	N/A	N/A	N/A
	UC-12-20	UHPC	Steel	Micro	0.12	2830	50	12.000%	Sand	2	OPC+FA	200	20	N/A	Gray-White	N/A	N/A	N/A	N/A	250	N/A	N/A	N/A
	UC-0-200	UHPC	N/A	N/A	N/A	N/A	N/A	0.000%	Sand	2	OPC+FA	200	200	0.200%	Gray-White	N/A	N/A	N/A	N/A	199.2	205.6	N/A	N/A
	UC-0-300	UHPC	N/A	N/A	N/A	N/A	N/A	0.000%	Sand	2	OPC+FA	200	300	1.500%	Gray-White	N/A	N/A	N/A	N/A	199.2	174.7	N/A	N/A
	UC-0-400	UHPC	N/A	N/A	N/A	N/A	N/A	0.000%	Sand	2	OPC+FA	200	400	2.500%	Gray-White	N/A	N/A	N/A	N/A	199.2	124.7	N/A	N/A
	UC-0-500	UHPC	N/A	N/A	N/A	N/A	N/A	0.000%	Sand	2	OPC+FA	200	500	3.000%	Gray-White	N/A	N/A	N/A	N/A	199.2	117.7	N/A	N/A
	UC-0-600	UHPC	N/A	N/A	N/A	N/A	N/A	0.000%	Sand	2	OPC+FA	200	600	3.800%	Gray-White	N/A	N/A	N/A	N/A	199.2	106.8	N/A	N/A
	UC-0-800	UHPC	N/A	N/A	N/A	N/A	N/A	0.000%	Sand	2	OPC+FA	200	800	4.800%	Gray-White	N/A	N/A	N/A	N/A	199.2	70.5	N/A	N/A
	UC-0-1000	UHPC	N/A	N/A	N/A	N/A	N/A	0.000%	Sand	2	OPC+FA	200	1000	4.800%	Grayish Brown	N/A	N/A	N/A	N/A	199.2	32.1	N/A	N/A
	UC-3-200	UHPC	Steel	Micro	0.12	2830	50	3.000%	Sand	2	OPC+FA	200	200	0.200%	Gray-White	N/A	N/A	N/A	N/A	213	219.8	N/A	N/A
	UC-3-300	UHPC	Steel	Micro	0.12	2830	50	3.000%	Sand	2	OPC+FA	200	300	1.500%	Gray-White	N/A	N/A	N/A	N/A	213	194.5	N/A	N/A
	UC-3-400	UHPC	Steel	Micro	0.12	2830	50	3.000%	Sand	2	OPC+FA	200	400	2.200%	Gray-White	N/A	N/A	N/A	N/A	213	140.8	N/A	N/A
	UC-3-500	UHPC	Steel	Micro	0.12	2830	50	3.000%	Sand	2	OPC+FA	200	500	2.800%	Gray-White	N/A	N/A	N/A	N/A	213	133.3	N/A	N/A
	UC-3-600	UHPC	Steel	Micro	0.12	2830	50	3.000%	Sand	2	OPC+FA	200	600	3.500%	Gray-White	N/A	Visible	N/A	N/A	213	131.2	N/A	N/A
	UC-3-800	UHPC	Steel	Micro	0.12	2830	50	3.000%	Sand	2	OPC+FA	200	800	4.000%	Gray-White	N/A	Visible	N/A	N/A	213	43.9	N/A	N/A
	UC-3-1000	UHPC	Steel	Micro	0.12	2830	50	3.000%	Sand	2	OPC+FA	200	1000	3.000%	Grayish Brown	N/A	pits	N/A	N/A	213	28.8	N/A	N/A
[28]	S0CA0	UHPGC	N/A	N/A	N/A	N/A	N/A	N/A	Natural	N/A	GGBS+FA+SF	20	700	6	Light Pale Brown	N/A	0.1	–	–	68	41	N/A	N/A
	S2CA0	UHPGC	Steel	Straight	0.20	2750	45	2.000%	Natural	N/A	GGBS+FA+SF	20	700	7	Light Brownish	N/A	0.08	–	–	78	39	N/A	N/A
	S0CA30	UHPGC	N/A	N/A	N/A	N/A	N/A	N/A	Natural	10	GGBS+FA+SF	20	700	7	Yellowish	N/A	0.12	–	–	61	38	N/A	N/A
	S2CA30	UHPGC	Steel	Straight	0.20	2750	45	2.000%	Natural	10	GGBS+FA+SF	20	700	7–8%	Brown	N/A	0.09	–	–	72	37	N/A	N/A
[29]	S0CA0	GPM	N/A	N/A	N/A	N/A	N/A	0.000%	Natural	3	GGBS+FA+SF	20	700	N/A	Light Brown	N/A	N/A	N/A	N/A	49.9	12.5	N/A	N/A
	S2CA0	GPM	Steel	Straight	0.27	2750	45	2.000%	Natural	3	GGBS+FA+SF	20	700	N/A	Light Brown	N/A	N/A	N/A	N/A	84.4	32.1	N/A	N/A
	S0CA30	GPC	N/A	N/A	N/A	N/A	N/A	0.000%	Crushed	10	GGBS+FA+SF	20	700	N/A	Yellowish	N/A	N/A	N/A	N/A	65.8	18.4	N/A	N/A
	S2CA30	GPC	Steel	Straight	0.27	2750	45	2.000%	Crushed	10	GGBS+FA+SF	20	700	N/A	Brown	N/A	N/A	N/A	N/A	76.6	26.8	N/A	N/A
[30]	MSFUHPC-1	UHPC	Steel	Straight	0.20	2800	65	1.000%	Natural	2	OPC+SF+FA+CSW+CE	105	800	10	Brown	N/A	Micro	27.5	20	135	95	N/A	N/A
	MSFUHPC-3	UHPC	Steel	Straight	0.20	2800	65	1.000%	Natural	2	OPC+SF+FA+CE	105	800	12	Yellow	N/A	Micro	25.5	18	120	87	N/A	N/A
[31]	PE05ST10	UHPC	Polypropylene + Steel	Straight	0.22	2800	59	1.000%	Natural	0.6	OPC+SF	No pre-drying	600	N/A	Brown	0.05	0.3	27.5	17.2	147	110	N/A	N/A
	PE10ST20	UHPC	Polypropylene + Steel	Straight	0.22	2800	59	2.000%	Natural	0.6	OPC+SF	No pre-drying	600	N/A	Dark Gray	0.06	0.4	24.2	15	147	127	N/A	N/A
	PE00ST00	UHPC	N/A	N/A	N/A	N/A	N/A	N/A	Natural	0.6	OPC+SF	No pre-drying	600	N/A	White	N/A	0.5	12.1	6.8	145	92	N/A	N/A
[32]	NF0	UHPC	N/A	N/A	N/A	N/A	N/A	N/A	Natural	N/A	OPC+SF	No pre-drying	800	9	White	N/A	N/A	151	60	11.4	5.2	N/A	N/A
	NSF2.0	UHPC	Steel	Straight	0.15	2500	66	2.000%	Natural	2	OPC+SF	No pre-drying	800	8	Light Brown	N/A	N/A	177.7	67	16.1	7.8	N/A	N/A
	NCF0.5	UHPC	N/A	N/A	N/A	N/A	N/A	N/A	Natural	0.5	OPC+SF	No pre-drying	800	7	Gray	N/A	N/A	164.9	62	17.5	7.5	N/A	N/A
	NCF0.25-SF2.0	UHPC	Steel	Straight	0.15	2500	66	2.000%	Natural	0.25	OPC+SF	No pre-drying	800	8	Brown	N/A	N/A	188	72	21	8.5	N/A	N/A
	RCF0.25-SF2.0	UHPC	Steel	Straight	0.15	2500	66	2.000%	Natural	0.25	OPC+SF	No pre-drying	800	9	Brown	N/A	N/A	180.9	70	20.3	8.2	N/A	N/A
[33]	U	UHPC	Steel	Straight	0.22	2800	64	2.000%	Natural	0.85	OPC+SF+FA+QP	20	400	N/A	Gray	0.05	0.4	25	0	132	0	N/A	N/A
	U-PET	UHPC	Polyethylene Terephthalate	Straight	0.22	2800	64	2.300%	Natural	0.85	OPC+SF+FA+QP	20	400	9.3	Gray	0.05	0.25	26.8	6.2	133	27	N/A	N/A
	U-PAN	UHPC	Polyacrylonitrile	Straight	0.22	2800	64	2.300%	Natural	0.85	OPC+SF+FA+QP	20	800	9.3	Yellow	0.05	0.3	26.5	6.5	134	28	N/A	N/A
	U-PVA	UHPC	Polyvinyl Alcohol	Straight	0.22	2800	64	2.300%	Natural	0.85	OPC+SF+FA+QP	20	800	9.3	Light Gray	0.05	0.28	26	6.8	132	30	N/A	N/A
	U-NY	UHPC	Nylon	Straight	0.22	2800	64	2.300%	Natural	0.85	OPC+SF+FA+QP	20	1050	8.7	Brown	0.05	0.2	28	7.5	135	32	N/A	N/A
	U-PP	UHPC	Polypropylene	Straight	0.22	2800	64	2.300%	Natural	0.85	OPC+SF+FA+QP	20	1050	9.1	Brown	0.05	0.18	27	7.2	135	33	N/A	N/A
[34]	C	UHPGC	N/A	N/A	N/A	N/A	N/A	N/A	Natural	20	FA+GGBS	25	800	9.38	Brick Red	0.05	0.4	5.2	1.6	60.1	11.7	3.1	0.8
	H-0.5-0.6	UHPGC	Polypropylene + Steel	Straight	0.21	2750	62	1.000%	Natural	20	FA+GGBS	25	800	9.2	Brick Red	0.05	0.32	5.8	1.9	69.4	13.5	3.4	1
	H-1.0-0.6	UHPGC	Polypropylene + Steel	Straight	0.21	2750	62	1.000%	Natural	20	FA+GGBS	25	800	9.3	Brick Red	0.05	0.3	6	2	72.8	14.5	3.5	1.1
	H-1.5-0.6	UHPGC	Polypropylene + Steel	Straight	0.21	2750	62	2.000%	Natural	20	FA+GGBS	25	800	9.4	Brick Red	0.05	0.25	6.2	2.1	74	14.4	3.7	0.8
	H-2.0-0.6	UHPGC	Polypropylene + Steel	Straight	0.21	2750	62	2.000%	Natural	20	FA+GGBS	25	800	9.5	Brick Red	0.05	0.35	6	1.8	71	13	3.6	0.9
[35]	MFC2	UHPC	Copper Coated	Straight	0.15	2500	85	2.000%	Natural	0.6	OPC+Microsilica	25	300	N/A	Gray	N/A	Micro	23	17	143	195	N/A	N/A
	MFC4	UHPC	Copper Coated	Straight	0.15	2500	85	4.000%	Natural	0.6	OPC+Microsilica	25	300	N/A	Gray	N/A	Micro	32	24	143	204	N/A	N/A
	MFC6	UHPC	Copper Coated	Straight	0.15	2500	85	6.000%	Natural	0.6	OPC+Microsilica	25	300	N/A	Gray	N/A	Micro	36	30	148	208	N/A	N/A
	MFC8	UHPC	Copper Coated	Straight	0.15	2500	85	8.000%	Natural	0.6	OPC+Microsilica	25	300	N/A	Gray	N/A	Micro	35	30	157	214	N/A	N/A
[36]	SFRC	UHPC	Steel	Hooked-end	0.90	1250	67	0.075%	Natural	20	OPC	105	800	10	Dark Gray	0.05	0.45	6.5	2.5	55	22	3	1
	SFRGPC (Na)	UHPGC	Steel	Hooked-end	0.90	1250	67	0.075%	Natural	20	FA	105	800	9	Brick Red	0.05	0.35	6.8	2.2	60	36	3.2	1.2
	SFRGPC (K)	UHPGC	Steel	Hooked-end	0.90	1250	67	0.075%	Natural	20	FA	105	800	9	Reddish Brown	0.05	0.3	6.8	2.4	58	35	3.2	2
[37]	SF1	UHPC	Polypropylene + Steel	Straight	0.20	2000	65	1.300%	Quartz	0.6	OPC+SF	105	600	5–7	Grayish White	0.05	0.40	14.03	11.36	133.4	125.3	N/A	N/A
	SF2	UHPC	Polypropylene + Steel	Straight	0.20	2000	65	2.300%	Quartz	0.6	OPC+SF	105	600	5–7	Grayish White	0.05	0.35	15.82	12.4	153.4	146.9	N/A	N/A
	SF3	UHPC	Polypropylene + Steel	Straight	0.20	2000	65	3.300%	Quartz	0.6	OPC+SF	105	600	5–7	Grayish White	0.05	0.30	19.38	14.32	173.6	159.8	N/A	N/A
[38]	GPL	UHPGC	N/A	N/A	N/A	N/A	N/A	N/A	Natural	N/A	GGBS+FA	20	600	5–12	Dark Light Gray	N/A	Micro	6.5	4.4	68	48	3.5	2.3
	GSF8	UHPGC	Steel	Straight	0.12	2800	67	1.000%	Natural	N/A	GGBS+FA	20	600	5–12	Dark Grayish Brown	N/A	Moderate	6.8	5.1	72	56	3.8	2.7
	GSF13	UHPGC	Steel	Straight	0.12	2800	108	1.000%	Natural	N/A	GGBS+FA	20	600	5–12	Dark Grayish Brown	N/A	Visible	7.2	5.3	74	58	3.9	2.8
[39]	S0CA0	UHPGC	N/A	N/A	N/A	N/A	N/A	N/A	Natural	N/A	GGBS+FA+SF	No pre-drying	900	4–8	Dark Gray	N/A	Visible	–	–	63	6	N/A	N/A
	S2CA0	UHPGC	Steel	Straight	0.20	2750	45	2.000%	Natural	N/A	GGBS+FA+SF	No pre-drying	900	4–8	Light Gray	N/A	Less spalling	–	–	68	7	N/A	N/A
	S0CA30	UHPGC	N/A	N/A	N/A	N/A	N/A	N/A	Natural	25	GGBS+FA+SF	No pre-drying	900	4–8	Grayish White	N/A	Visible	–	–	70	8	N/A	N/A
	S2CA30	UHPGC	Steel	Straight	0.20	2750	45	2.000%	Natural	25	GGBS+FA+SF	No pre-drying	900	4–8	Grayish White	N/A	Spalling	–	–	73	7	N/A	N/A
[40]	REF	UHPC	N/A	N/A	N/A	N/A	N/A	N/A	Natural	1.25	OPC+SF+FA	105	800	7.9	Whitish	N/A	Visible	14.2	4.8	168	58	9.1	2.6
	SF	UHPC	Steel	Straight	0.22	2800	64	2.000%	Natural	1.25	OPC+SF+FA	105	800	8.1	Brownish	0.05	0.4	16.5	6.3	177	67	9.8	3.4
	MF	UHPC	Polyvinyl Alcohol	Micro	0.04	1600	N/A	1.000%	Natural	1.25	OPC+SF+FA	105	800	8.2	Light Brown	0.05	0.35	15.8	6.5	170	70	9.3	3.6
	HF	UHPC	Polyvinyl Alcohol + Steel	Micro	0.22	2800	64	2.500%	Natural	1.25	OPC+SF+FA	105	800	8.3	Brown	0.05	0.3	18.6	7.2	182	71	10.4	4.2
[41]	S2.0	UHPC	Steel	Micro	0.30	2850	83	2.000%	Natural	1	OPC+GGBS+SF+QP	No pre-drying	800	5%	White	N/A	Micro	9.3	2.3	165	58	8.8	2.6
	S1.5M0.5	UHPC	Steel	Micro	0.30	2850	74	2.000%	Natural	1	OPC+GGBS+SF+QP	No pre-drying	800	5%	White	N/A	Visible	8.3	2.4	165	59	8.3	2.4
	S0.5M1.5	UHPC	Steel	Micro	0.30	2850	74	2.000%	Natural	1	OPC+GGBS+SF+QP	No pre-drying	800	5%	Light Gray	N/A	Visible	9.3	1.8	170	60	9.3	1.8
	M2.0	UHPC	Steel	Micro	0.20	2850	65	2.000%	Natural	1	OPC+GGBS+SF+QP	No pre-drying	800	5%	Light Brown	N/A	Visible	8.8	2.6	160	60	8.8	2.6
[42]	SCC	SCC	N/A	N/A	N/A	N/A	N/A	0%	Dolomite + River Sand	9.5	OPC+SF	No pre-drying	800	9	Reddish Brown	N/A	Spalling/Destroyed	6.4	0	50.2	4.6	4.1	0
	SCC-L1	SCC	Glass	Chopped	0.013	1700	1000	1%	Dolomite + River Sand	9.5	OPC+SF	No pre-drying	800	7.6	Reddish Brown	N/A	Spalling/Destroyed	8.6	0	51.8	6.4	4.5	0
	SCC-L2	SCC	Glass	Chopped	0.013	1700	1000	1%	Dolomite + River Sand	9.5	OPC+SF	No pre-drying	800	8	Reddish Brown	N/A	Spalling/Destroyed	9.8	0	52.4	7.2	4.9	0
	SCC-S1	SCC	Glass	Chopped	0.013	1700	462	1%	Dolomite + River Sand	9.5	OPC+SF	No pre-drying	800	8.2	Reddish Brown	N/A	Spalling/Destroyed	8.6	0	50.4	6.2	4.6	0
	SCC-S2	SCC	Glass	Chopped	0.013	1700	462	1%	Dolomite + River Sand	9.5	OPC+SF	No pre-drying	800	8.2	Reddish Brown	N/A	Spalling/Destroyed	9	0	51	5.8	4.7	0
[43]	1SF0	CRC (C35)	N/A	N/A	N/A	N/A	N/A	0%	Stone + Sand + Rubber	20	OPC	20	600	7.7	N/A	N/A	Spalling/Cracked	N/A	N/A	38.7	30.3	2.9	1.9
	1SF0.5	CRC (C35)	Steel	Hooked-end	0.55	1345	64	1%	Stone + Sand + Rubber	20	OPC	20	600	7.9	N/A	N/A	Visible Cracks	N/A	N/A	42.8	31.7	3.3	2.5
	1SF1	CRC (C35)	Steel	Hooked-end	0.55	1345	64	1%	Stone + Sand + Rubber	20	OPC	20	600	9.3	N/A	N/A	Visible Cracks	N/A	N/A	44.6	35.5	4.9	3.3
	1SF1.5	CRC (C35)	Steel	Hooked-end	0.55	1345	64	2%	Stone + Sand + Rubber	20	OPC	20	600	8.1	N/A	N/A	Visible Cracks	N/A	N/A	40	30.7	4.4	3.4
	1SF1NS1	CRC (C35)	Steel	Hooked-end	0.55	1345	64	1%	Stone + Sand + Rubber	20	OPC + 1% Nano-Silica	20	600	9.5	N/A	N/A	Minor Cracks	N/A	N/A	47.1	39	5.1	3.5
	1SF1NS2	CRC (C35)	Steel	Hooked-end	0.55	1345	64	1%	Stone + Sand + Rubber	20	OPC + 2% Nano-Silica	20	600	8.6	N/A	N/A	Minor Cracks	N/A	N/A	47.3	47.3	5.2	3.2
	2SF0	CRC (C45)	N/A	N/A	N/A	N/A	N/A	0%	Stone + Sand + Rubber	20	OPC	20	600	10.4	N/A	N/A	Spalling/Cracked	N/A	N/A	51.3	32.9	3.3	1.5
	2SF0.5	CRC (C45)	Steel	Hooked-end	0.55	1345	64	1%	Stone + Sand + Rubber	20	OPC	20	600	8.1	N/A	N/A	Visible Cracks	N/A	N/A	52.8	36.6	3.6	2.3
	2SF1	CRC (C45)	Steel	Hooked-end	0.55	1345	64	1%	Stone + Sand + Rubber	20	OPC	20	600	8.4	N/A	N/A	Visible Cracks	N/A	N/A	53.5	39.9	4.4	3.6
	2SF1.5	CRC (C45)	Steel	Hooked-end	0.55	1345	64	2%	Stone + Sand + Rubber	20	OPC	20	600	11.9	N/A	N/A	Visible Cracks	N/A	N/A	54.3	40.4	5	3.6
	2SF1NS1	CRC (C45)	Steel	Hooked-end	0.55	1345	64	1%	Stone + Sand + Rubber	20	OPC + 1% Nano-Silica	20	600	9.6	N/A	N/A	Minor Cracks	N/A	N/A	54.9	51.7	5.1	3.7
	2SF1NS2	CRC (C45)	Steel	Hooked-end	0.55	1345	64	1%	Stone + Sand + Rubber	20	OPC + 2% Nano-Silica	20	600	7.8	N/A	N/A	Minor Cracks	N/A	N/A	56.1	51.8	4.9	3.4
[44]	C30	LWC	N/A	N/A	N/A	N/A	N/A	0%	Sludge LWA + Sand	N/A	OPC+Slag	100	800	N/A	N/A	N/A	N/A	5.7	2.1	34.4	16.5	N/A	N/A
	E30-S	LWC	Steel	Wavy	0.8	2000	37.5	1%	Sludge LWA + Sand	N/A	OPC+Slag	100	800	N/A	N/A	N/A	N/A	5.4	2.5	35.4	19.1	N/A	N/A
	C50	LWC	N/A	N/A	N/A	N/A	N/A	0%	Sludge LWA + Sand	N/A	OPC+Slag	100	800	N/A	N/A	N/A	N/A	5.5	2.9	60.8	33.2	N/A	N/A
	E50-S	LWC	Steel	Wavy	0.8	2000	37.5	1%	Sludge LWA + Sand	N/A	OPC+Slag	100	800	N/A	N/A	N/A	N/A	5.8	3.2	54.1	29.6	N/A	N/A
	E50-P	LWC	Polypropylene	Monofilament	0.05	300	240	0%	Sludge LWA + Sand	N/A	OPC+Slag	100	800	N/A	N/A	N/A	N/A	5.2	2.5	54.7	28.5	N/A	N/A
	E50-M	LWC	Steel + Polypropylene	Wavy + Monofilament	0.8	2000	37.5	1%	Sludge LWA + Sand	N/A	OPC+Slag	100	800	N/A	N/A	N/A	N/A	6.7	3.9	52.8	32.8	N/A	N/A
[45]	NC	SCC	N/A	N/A	N/A	N/A	N/A	0%	Limestone + River Sand	10	OPC+Fly Ash	20	600	8-10	N/A	N/A	Severe Spalling	5.73	0.95	65.1	47.7	N/A	N/A
	PF1	SCC	Polypropylene	Monofilament	0.0305	450	623	0%	Limestone + River Sand	10	OPC+Fly Ash	20	600	8-10	N/A	N/A	No Spalling	6.08	1.06	57.1	38.6	N/A	N/A
	PF2	SCC	Polypropylene	Monofilament	0.0305	450	623	0%	Limestone + River Sand	10	OPC+Fly Ash	20	600	8-10	N/A	N/A	No Spalling	5.48	3.11	54.8	38	N/A	N/A
	SF20	SCC	Steel	Hooked-end	0.625	1150	80	0%	Limestone + River Sand	10	OPC+Fly Ash	20	600	8-10	N/A	N/A	Explosive Spalling	7.6	2.81	68.4	47.8	N/A	N/A
	SF40	SCC	Steel	Hooked-end	0.625	1150	80	1%	Limestone + River Sand	10	OPC+Fly Ash	20	600	8-10	N/A	N/A	Explosive Spalling	8.43	3.88	67.6	48.7	N/A	N/A
	SF50	SCC	Steel	Hooked-end	0.625	1150	80	1%	Limestone + River Sand	10	OPC+Fly Ash	20	600	8-10	N/A	N/A	Minor Spalling	8.14	4.95	68.5	52.7	N/A	N/A
	SPF201	SCC	Steel + Polypropylene	Hooked + Mono	0.625	1150	80	0%	Limestone + River Sand	10	OPC+Fly Ash	20	600	8-10	N/A	N/A	No Spalling	6.75	1.66	62.4	41.1	N/A	N/A
[46]	UP	UHPC	Polypropylene	Monofilament	0.031	510	386	0%	Basalt	10	OPC+SF+FA	90	800	~9.0	Light Yellow	N/A	Intact (No Spalling)	N/A	N/A	120	36	N/A	N/A
	UN	UHPC	Polyacrylonitrile	Monofilament	0.012	625	1000	0%	Basalt	10	OPC+SF+FA	90	800	~7.5	Light Yellow	N/A	Exploded/Spalled	N/A	N/A	110	N/A (Exploded)	N/A	N/A
	US	UHPC	Steel	Straight	0.2	2100	60	1%	Basalt	10	OPC+SF+FA	90	800	~3.6	Light Yellow	N/A	Intact (No Spalling)	N/A	N/A	130	40	N/A	N/A
	SP	UHPC	Steel + Polypropylene	Hybrid	Various	Various	Various	1%	Basalt	10	OPC+SF+FA	90	800	~8.0	Light Yellow	N/A	Intact (No Spalling)	N/A	N/A	135	40	N/A	N/A
	SN	UHPC	Steel + Polyacrylonitrile	Hybrid	Various	Various	Various	1%	Basalt	10	OPC+SF+FA	90	800	~7.5	Light Yellow	N/A	Intact (No Spalling)	N/A	N/A	125	38	N/A	N/A
Note	Aggregate type: fine quartz/natural sand to crushed stone, dolomite, limestone, basalt, rubber crumb, sludge-based lightweight aggregate Max aggregate size: 0.25–25 mm Fibre diameter: 0.012–0.90 mm Fibre tensile strength: 300–2850 MPa Fibre aspect ratio (L/D): 37.5–1000 Fibre volume fraction: 0–12% Binder type: OPC-based, OPC+Slag, GGBS/FA-based (geopolymer) Pre-treatment/drying temperature: 20–200 °C (or no pre-drying) Exposure temperature (T): 20–1050 °C Ambient compressive strength (*f_c_*): ~34–250 MPa Residual compressive strength (*f*′*_c_*): 0–~220 MPa Ambient flexural/tensile strength (*f_t_*/*f_m_*): ~2.9–10.4 MPa Residual flexural/tensile strength (*f*′*_t_*/*f*′*_m_*): 0–4.2 MPa Spalling response: none to severe/explosive spalling																						

$T_{pre}$, Pre-Drying Temperature, °C; *T*, Maximum Attack Temperature (°C); $T_{l}$, Thermal Mass Loss (%); $C_{a}$, Sample Colour after Attack; ${MC}_{b}$, Maximum Crack Width Before Attack, mm; ${MC}_{a}$, Maximum Crack Width After Attack, mm; $f_{m}$, Flexural Strength Before Attack (MPa); ${f\prime }_{m}$, Flexural Strength After Attack (MPa); $f_{c}$, Uniaxial Compressive Strength Before Attack (MPa); ${f\prime }_{c}$, Uniaxial Compressive Strength After Attack (MPa); $f_{t}$, Splitting Tensile Strength Before Attack (MPa); and ${f\prime }_{t}$, Splitting Tensile Strength After Attack (MPa).
